# Comparative Effectiveness of Postdischarge Smoking Cessation Interventions for Hospital Patients: The Helping HAND 4 Randomized Clinical Trial

**DOI:** 10.1001/jamainternmed.2022.2300

**Published:** 2022-06-27

**Authors:** Nancy A. Rigotti, Yuchiao Chang, Esa M. Davis, Susan Regan, Douglas E. Levy, Thomas Ylioja, Jennifer H. K. Kelley, Anna E. Notier, Karen Gilliam, Antoine B. Douaihy, Daniel E. Singer, Hilary A. Tindle

**Affiliations:** 1 Tobacco Research & Treatment Center, Massachusetts General Hospital, Boston; 2 Division of General Internal Medicine, Department of Medicine, Massachusetts General Hospital, Boston; 3 Health Policy Research Center, Mongan Institute, Massachusetts General Hospital, Boston; 4 Harvard Medical School, Boston, Massachusetts; 5 University of Pittsburgh School of Medicine, Pittsburgh, Pennsylvania; 6 University of Pittsburgh Medical Center, Pittsburgh, Pennsylvania; 7 National Jewish Health, Denver, Colorado; 8 Vanderbilt University Medical Center, Nashville, Tennessee; 9 Geriatric Research Education and Clinical Centers, Veterans Affairs Tennessee Valley Healthcare System, Nashville

## Abstract

**Importance:**

Smoking cessation interventions for hospitalized patients must continue after discharge to improve long-term tobacco abstinence. How health systems can best deliver postdischarge tobacco treatment is uncertain.

**Objective:**

To determine if health system–based tobacco cessation treatment after hospital discharge produces more long-term tobacco abstinence than referral to a community-based quitline.

**Design, Setting, and Participants:**

This randomized clinical trial was conducted September 2018 to November 2020 in 3 hospitals in Massachusetts, Pennsylvania, and Tennessee. Cigarette smokers admitted to a study hospital who received brief in-hospital tobacco treatment and wanted to quit smoking were recruited for participation and randomized for postdischarge treatment to health system–based Transitional Tobacco Care Management (TTCM) or electronic referral to a community-based quitline (QL). Both multicomponent interventions offered smoking cessation counseling and nicotine replacement therapy (NRT) for up to 3 months. Data were analyzed from February 1, 2021, to April 25, 2022.

**Interventions:**

TTCM provided 8 weeks of NRT at discharge and 7 automated calls with a hospital-based counselor call-back option. The QL intervention sent referrals from the hospital electronic health record to the state quitline, which offered 5 counseling calls and an NRT sample.

**Main Outcomes and Measures:**

The main outcome was biochemically verified past 7-day tobacco abstinence at 6 months. Self-reported point-prevalence and continuous tobacco abstinence and tobacco treatment utilization were assessed 1, 3, and 6 months after discharge.

**Results:**

A total of 1409 participants (mean [SD] age, 51.7 [12.6] years; 784 [55.6%] women; mean [SD] 16.4 [10.6] cigarettes/day) were recruited, including 706 randomized to TTCM and 703 randomized to QL. Participants were comparable at baseline, including 216 Black participants (15.3%), 82 Hispanic participants (5.8%), and 1089 White participants (77.3%). At 1 and 3 months after discharge, more TTCM participants than QL participants used cessation counseling (1 month: 245 participants [34.7%] vs 154 participants [21.9%]; 3 months: 248 participants [35.1%] vs 123 participants [17.5%]; *P* < .001) and pharmacotherapy (1 month: 455 participants [64.4%] vs 324 participants [46.1%]; 3 months: 367 participants [52.0%] vs 264 participants [37.6%]; *P* < .001). More TTCM than QL participants reported continuous abstinence for 3 months (RR, 1.30; 95% CI, 1.06-1.58) and point-prevalence abstinence at 1 month (RR, 1.22; 95% CI, 1.08-1.35) and 3 months (RR, 1.23; 95% CI, 1.09-1.37) but not at 6 months (RR, 1.14; 95% CI, 0.99-1.29). The primary outcome, biochemically verified point-prevalence abstinence at 6 months, was not statistically significantly different between groups (19.9% vs 16.9%; RR, 1.18; 95% CI, 0.92-1.50).

**Conclusions and Relevance:**

In this randomized clinical trial, biochemically verified tobacco abstinence rates were not significantly different between groups at the 6-month follow-up. However, the health system–based model was superior to the community-based quitline model throughout the 3 months of active treatment. A longer duration of postdischarge treatment may sustain the superiority of the health system–based model.

**Trial Registration:**

ClinicalTrials.gov Identifier: NCT03603496

## Key Points

**Question:**

How can health systems most effectively provide smoking cessation treatment to patients after hospital discharge to promote long-term tobacco abstinence?

**Findings:**

This randomized clinical trial of 1409 hospitalized smokers compared 2 models for delivering tobacco cessation treatment after discharge: health system–based care or referral to a community-based quitline. Health system–based care produced better treatment use and more tobacco abstinence throughout the 3 months of treatment but not 6 months after discharge.

**Meaning:**

These findings suggest that continuing treatment beyond 3 months might have sustained end-of-treatment superiority of the health system–based model.

## Teaser

This randomized clinical trial compares health system–based smoking cessation treatment with referral to community-based quitline services after hospital discharge.

## Introduction

Cigarette smoking remains the leading preventable cause of death in the United States,^1^ with 480 000 smoking-attributable deaths annually.^2^ In 2019, 14% of US adults smoked cigarettes.^3^ More than 3.2 million adults who smoke are hospitalized annually.^4^ Hospital admission offers them a unique opportunity to quit smoking.^5^ Hospital no-smoking policies require temporary tobacco abstinence that patients who smoke can use to initiate cessation.^5^ The illness prompting admission, especially if smoking-related, can enhance motivation to quit, and nicotine replacement therapy (NRT) that is often used to relieve nicotine withdrawal while in the hospital gives adults who smoke an opportunity to sample a cessation medication.

Strong evidence supports the long-term effectiveness of smoking cessation interventions that begin in the hospital, but only if they are sustained after discharge. In a meta-analysis of 50 randomized clinical trials,^5^ cessation counseling that began in the hospital was associated with increasing tobacco abstinence rates at 6 months after discharge by 37%, with additional benefit associated with adding NRT to counseling. However, the association was observed only if treatment continued for at least 1 month after discharge. Applying this evidence, clinical guidelines urge health care clinicians to address tobacco use with all hospitalized patients who use tobacco.^6,7^ National Hospital Quality Measures, adopted by the Joint Commission and Medicare, mandate documenting smoking status on admission and offering smoking cessation counseling and medication to tobacco users during hospitalization and at discharge.^8^

In practice, sustaining smoking cessation treatment during the inpatient to outpatient care transition is a challenge for health care systems.^5,9^ Two models have emerged to meet the challenge, but the optimal approach is uncertain. One model keeps tobacco treatment based in the health care system, resembling the management of other chronic medical conditions. Patients receive cessation medication at discharge and 3 months of telephone-based behavioral support and medication management from a tobacco treatment specialist who coordinates care with the patients’ clinical team.^10,11^ We demonstrated the effectiveness of this model over standard care in a randomized trial that increased tobacco abstinence rates by 71% at 6 months (26% vs 15%; *P* < .01).^10^

An alternative care model transfers postdischarge treatment from the hospital to a community-based resource, the national system of telephone quitlines funded by state public health departments.^12,13,14,15^ Quitline coaches provide free behavioral cessation counseling, and most quitlines offer free mailed samples of NRT to eligible callers.^12,15^ Quitlines are attractive to hospitals because their services have no cost to the hospital, although some state quitlines do not offer multisession counseling to all callers. In practice, quitlines receive few postdischarge referrals and have had difficulty engaging recently hospitalized patients who smoke in treatment and increasing cessation rates compared with standard care.^13,14,16,17^ Technology now allows hospital electronic health records (EHR) to send a referral directly to a quitline using a secure Health Insurance Portability and Accountability Act (HIPAA)–adherent connection. Upon receiving a referral, quitline staff call the patient to offer cessation services.^16,18^ The effectiveness of this model for engaging patients or improving cessation rates after hospital discharge has not been tested.

This multisite randomized clinical trial is the first trial, to our knowledge, to compare these 2 models for delivering tobacco cessation services after hospital discharge. We hypothesized that providing treatment through the health care system would generate better treatment engagement and more smoking cessation at 6 months after hospital discharge than referral to a community-based quitline. The rationale was that health system-based care leverages patients’ familiarity and trust in existing caregivers and permits tobacco counselors to manage cessation medications and coordinate care directly with a patient’s clinical team.

## Methods

The 3-site Helping HAND 4 (Hospital-Initiated Assistance for Nicotine Dependence) randomized clinical trial compared 2 strategies to improve smoking cessation rates after hospital discharge. Institutional review boards at each hospital approved the study, and all participants provided oral informed consent. A detailed study protocol is available in Supplement 1 and has been published elsewhere.^19^

### Setting and Participants

The trial was conducted at Massachusetts General Hospital (Boston, Massachusetts), University of Pittsburgh Medical Center (Pittsburgh, Pennsylvania), and Vanderbilt University Medical Center (Nashville, Tennessee). Adults (age ≥18 years) admitted to these hospitals were eligible for inclusion if they smoked cigarettes daily in the month before admission, received smoking cessation counseling in the hospital, planned to try to quit smoking after discharge and agreed to accept an NRT sample at discharge. Patients were excluded if they lacked a home address or reliable telephone access, had low English proficiency, had psychiatric or cognitive impairment that precluded informed consent or participation in counseling, were admitted to obstetric or psychiatric units, or had less than 1 year of life expectancy.

### Recruitment and Assignment to Condition

Each hospital documented a patient’s smoking status at admission. A Tobacco Treatment Service counselor attempted to visit every patient who smoked to offer cessation assistance and screen for eligibility. Research staff confirmed eligibility, obtained oral informed consent, conducted the baseline assessment, and randomly assigned participants to study group. Randomization was stratified by study site, admitting diagnosis (cardiac vs other), and cigarettes per day (≥10 or <10) using permuted blocks of size 4. Participants were randomly assigned (1:1) to health system–based Transitional Tobacco Care Management (TTCM) or community-based quitline electronic referral (QL) using a computer-generated randomization scheme and the REDCap randomization module (Vanderbilt University). Investigators, but neither participants nor study staff, were blinded to study condition.

### Interventions

All participants received smoking cessation counseling, were offered NRT for nicotine withdrawal relief in the hospital. Participants were advised to continue treatment after discharge.

#### Transitional Tobacco Care Management

The TTCM health care system–based multicomponent intervention was adapted from our previous model.^10,11,19,20,21,22^ At discharge, participants received a free 8-week supply of their choice of NRT patch, gum, or lozenge (alone or in combination). Postdischarge counseling included 7 automated phone calls using interactive voice response (IVR) technology. At 3 days and 2, 4, 6, 8, 10, and 12 weeks after discharge, IVR calls monitored smoking status, encouraged medication adherence, supported cessation efforts, and offered a return call from a health system–based tobacco counselor who provided 5 to 10 minutes of behavioral counseling, promoted medication adherence and coordinated medications with the outpatient clinician.^11^ At 12 weeks, the counselor sent an EHR message to transition tobacco treatment responsibility to the participant’s outpatient clinician. The message summarized tobacco treatment provided, current smoking status, and future treatment recommendations, including advice to contact the participant proactively to offer assistance.

#### Quitline Electronic Referral

Postdischarge tobacco cessation care was transferred to the state quitline at discharge using a secure HIPAA-adherent link from the EHR.^16,18,23^ Quitline staff called the participant to offer telephone counseling calls from a trained coach and a free NRT sample to eligible callers. Each state’s contract with the quitline operator determined how many counseling sessions (typically 5 over 3 months) and weeks of NRT sample were offered.^19^ The study protocol stipulated bidirectional electronic referral to allow quitline reports of the referral outcome to return to the participant’s EHR. However, technological barriers allowed only unidirectional electronic referral at Massachusetts General Hospital and University of Pittsburgh Medical Center.^19^

### Assessments

Baseline measures, obtained by in-person interview, included sociodemographic characteristics (age, sex, self-reported race and ethnicity, education); past 7-day use of tobacco products (cigarettes, electronic cigarettes, other products); nicotine dependence^24^; previous quit attempts and cessation treatments used; intention to quit after discharge; perceived importance of, confidence in, and social support for quitting (each assessed using 10-point Likert scales); alcohol use (assessed using the Alcohol Use Disorders Identification Test^25^); past 30-day use of marijuana, cocaine, opioids, stimulants, or injection drugs; and past 2-week anxiety and depression symptoms (assessed using the 7-item Generalized Anxiety Disorder Assessment^26^ and the 8-item Patient Health Questionnaire depression scale^27^). Race and ethnicity were categorized as Asian or Pacific Islander; Black, non-Hispanic; Hispanic; White, non-Hispanic; and other or unknown (including American Indian, multiple races, and those who identified as other race or ethnicity) and were included to assess the generalizability of the findings and determine if the effectiveness of the interventions varied by these factors. Discharge diagnoses, health insurance, and length of stay were obtained from the EHR. Interactive voice response and quitline records provided information about intervention delivery.

Participants were contacted by email or text message at 1, 3, and 6 months after discharge to complete web-based follow-up surveys. Telephone or mailed surveys were administered as backup. Participants received $20 per completed survey. Surveys assessed postdischarge use of tobacco products (continuous and past 7-day abstinence), tobacco cessation pharmacotherapy, and behavioral support.

### Outcome Measures

The primary outcome measure was biochemically validated past 7-day abstinence from cigarettes and other conventional tobacco products at the 6-month follow-up. As guidelines for measuring abstinence in clinical trials recommend,^28^ abstinence was defined as no tobacco product use except e-cigarettes. A sensitivity analysis calculated abstinence not allowing e-cigarette use. Verified abstinence required self-reported 7-day abstinence at 6 months plus either a mailed saliva sample with a cotinine concentration no more than 10 μg/L (to convert to nanomoles per liter, multiply by 5.675),^29^ or if NRT or e-cigarette was in use, an expired air sample with carbon monoxide (co) no more than 9 ppm.^30^ Carbon monoxide samples were obtained in person or remotely by mailing a personalized co testing device (iCO Smokerlyzer; CoVita) to participants who could not be seen in person owing to distance or COVID-19 restrictions. Participants received $50 for submitting a saliva sample or co measurement. Payment was increased to $150 in January 2020 to promote sample return. Secondary tobacco cessation outcomes included self-reported past 7-day tobacco and continuous abstinence at each follow up point (1, 3, and 6 months).

Participants’ engagement in postdischarge smoking cessation treatment was assessed as the proportion who reported current use of either pharmacotherapy or behavioral support at each follow-up. Quitline and study records were used to measure the delivery of study intervention components (phone calls and medications).

### Statistical Analysis

A sample of 1350 participants (675 participants per group) was planned to detect a 6.5% difference (23.0% vs 16.5%) in the primary outcome measure with 84% power and a 2-sided *P* < .05.^19^ Analyses used an intention-to-treat approach. Data from the 3 sites were combined for analysis after Cochran-Mantel-Haenzel tests confirmed that intervention effects were not heterogeneous across sites. χ^2^ tests and *t* tests were used to assess the effect of study group on primary and secondary outcomes. Participants who self-reported smoking or whose cotinine or co measures exceeded the cutoffs were coded as smokers. Participants who were lost to follow-up or reported not smoking but did not provide verification of self-report were coded as missing. We used 2-stage multiple imputation techniques to estimate missing smoking outcomes while accounting for uncertainty from missing data.^31^ We imputed missing survey data in the first-stage model and imputed the missing data from biochemical samples in the second-stage model. Multiple imputation models included variables associated with missingness and with cessation outcomes.^32^ We conducted 2 sensitivity analyses: respondents only and counting participants with missing data as smokers. Longitudinal analyses using generalized estimating equations techniques were used to assess the overall impact of study group by including data from all follow-up times. Self-reported duration of continuous tobacco abstinence after discharge was estimated using Kaplan-Meier curves and compared using a log-rank test. Prespecified subgroup analyses were conducted to test whether the intervention effect varied by site, age, sex, race and ethnicity, nicotine dependence, years smoked, depression and anxiety symptoms, smoking-related discharge diagnosis (vs other), and duration of hospitalization. A 2-sided significance level of *P* = .05 was considered for the primary outcome and a 2-sided significance level of *P* = .017 was considered for outcomes measured at 3 time points. Analyses were conducted using SAS statistical software version 9.4 (SAS Institute). Data were analyzed from February 1, 2021, to April 25, 2022.

## Results

**Figure 1.**
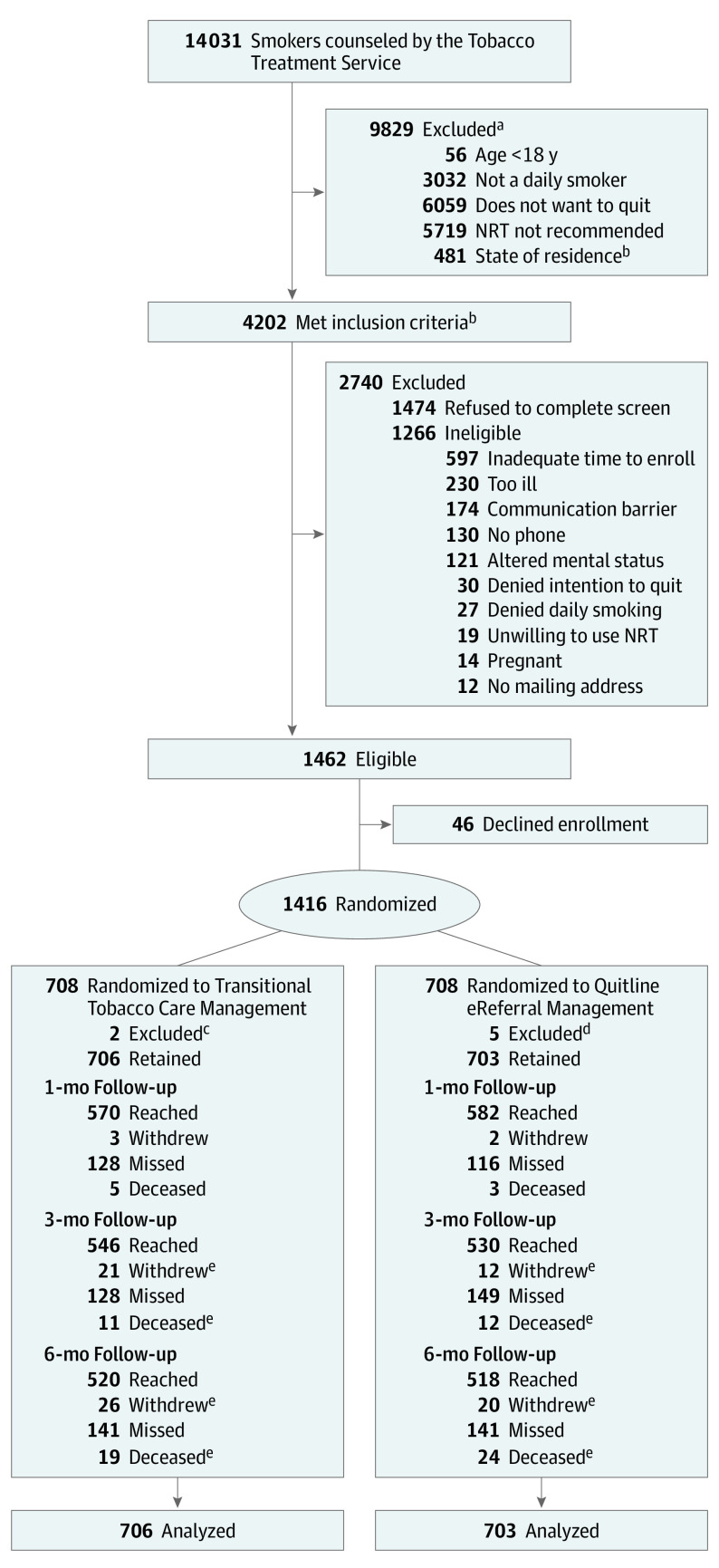
Participant Recruitment Flowchart: Helping HAND 4 Randomized Clinical Trial ^a^Patients may have had more than 1 reason for exclusion. ^b^Inclusion criteria were age at least 18 years, daily smoker, intends to quit, eligible for nicotine replacement therapy use after discharge (decision of hospital physician), and resides in a state served by participating quitline provider (Massachusetts, New Hampshire, Rhode Island, Vermont, Pennsylvania, Alaska, Colorado, Idaho, Iowa, Kentucky, Michigan, Missouri, Nevada, North Dakota, Ohio, Wyoming, and Tennessee). ^c^Excluded after randomization (because they were found to be ineligible). ^d^Excluded after randomization, including 4 participants who withdrew before discharge and 1 participant found to be ineligible. ^e^The numbers of patients who withdrew and died are cumulative.

From September 10, 2018, to March 9, 2020, 14 031 patients who smoked were counseled at the participating 3 hospitals, and 4202 (30%) met study inclusion criteria (Figure 1). Of these, 1462 patients (35%) were eligible. Figure 1 shows the most common reasons for ineligibility. A total of 1416 participants (97% of those eligible; 52% of those screened) enrolled and were randomly assigned to TTCM (708 participants) or QL (708 participants). Seven participants were excluded after randomization (Figure 1), leaving 1409 participants (mean [SD] age, 51.7 [12.6] years; 784 [55.6%] women), including 706 TTCM participants and 703 QL participants, for analysis. Follow-up assessment completion rates were 82% at 1 month, 76% at 3 months, and 74% at 6 months and did not differ by group. Patients lost to follow-up were younger, had less education and were less likely to have a smoking-related discharge diagnosis. By the end of follow-up data collection in November 2020, 43 participants (3%) had died, including 19 participants in the TTCM group and 24 participants in the QL group (eTable in Supplement 2).

Baseline characteristics and hospital course were comparable between study groups (Table 1). Among all participants, 444 primary discharge diagnoses (31%) were smoking-related.^2^

**Table 1.** Baseline Characteristics of Study Participants by Treatment Group

Characteristic	No. (%)	
	TTCM (n = 706)	QL (n = 703)
Age, mean (SD), y	51.5 (12.8)	51.9 (12.3)
Sex		
Women	391 (55.4)	393 (55.9)
Men	315 (44.6)	310 (44.1)
Race and ethnicity		
Asian or Pacific Islander	4 (0.6)	3 (0.4)
Black non-Hispanic	116 (16.4)	100 (14.2)
Hispanic	39 (5.5)	43 (6.1)
White non-Hispanic	539 (76.3)	550 (78.2)
Other or unknown^a^	8 (1.1)	7 (1.0)
Education		
≤High school or GED	388 (55.0)	414 (59.1)
Some college	97 (13.7)	86 (12.3)
≥College graduate	221 (31.3)	201 (28.7)
Health insurance		
Commercial	174 (24.6)	178 (25.3)
Medicare	183 (25.9)	179 (25.5)
Medicaid	219 (31.0)	217 (30.9)
Other	130 (18.4)	129 (18.3)
Tobacco use		
Cigarettes per day, past 30 d, mean (SD)	16 (10.0)	17 (11.0)
Past 30-d use of e-cigarettes	59 (8.4)	59 (8.4)
Past 30-d use of other tobacco product	49 (7.0)	58 (8.3)
Smoke first cigarette within 30 min of waking	547 (77.6)	563 (80.1)
Heaviness of smoking index, mean (SD)^b^	2.9 (1.5)	3 (1.5)
Smoking duration, mean (SD), y	34 (14.0)	35 (13.0)
Live with a smoker	324 (49.1)	338 (51.4)
Quitting history		
Past-year quit attempt (>24 h)	369 (52.3)	399 (56.8)
Prior use of cessation medications		
NRT	377 (53.6)	382 (54.4)
Bupropion	120 (17.0)	138 (19.7)
Varenicline	165 (23.5)	166 (23.7)
None	275 (39.1)	263 (37.5)
Prior use of smoking cessation counseling^c^	76 (10.9)	79 (11.3)
Importance to quit now score, mean (SD)^d^	9.4 (1.2)	9.5 (1.2)
Confidence to resist urge to smoke score, mean (SD)	7.8 (2.1)	7.8 (2.1)
Social support for quitting score, mean (SD)^d^	8.7 (2.5)	8.7 (2.5)
Comorbidities		
Depression symptoms, mean (SD)^e^	9.3 (6.5)	9.6 (6.5)
Anxiety symptoms, mean (SD)^f^	10.4 (6.2)	11 (6.2)
Alcohol use disorder^g^	166 (23.6)	164 (23.4)
Past 6-mo use		
Marijuana	193 (27.4)	222 (31.6)
Cocaine	32 (4.5)	44 (6.3)
Opioids not prescribed by a doctor	30 (4.3)	38 (5.4)
**Hospital course**		
Length of stay, median (IQR), d	5 (3-7)	4 (2-7)
Primary discharge diagnosis		
Any smoking-related disease^h^	207 (29.3)	233 (33.1)
*ICD-10* diagnosis groups, %		
Circulatory^i^	199 (28.2)	201 (28.6)
Injury, poisoning	115 (16.3)	106 (15.1)
Digestive	58 (8.2)	76 (10.8)
Respiratory	54 (7.6)	58 (8.3)
Musculoskeletal	52 (7.4)	49 (7.0)
Neoplasms	48 (6.8)	44 (6.3)
Signs, symptoms, ill-defined conditions	44 (6.2)	28 (4.0)
Endocrine, metabolic	27 (3.8)	28 (4.0)
Nervous system	30 (4.2)	26 (3.7)
Other	79 (11.2)	87 (12.4)

Abbreviations: GED, General Educational Development; *ICD-10*, *International Statistical Classification of Diseases and Related Health Problems, Tenth Revision*; NRT, nicotine replacement therapy; QL, quitline electronic referral; TTCM, Transitional Tobacco Care Management.

^a^ Includes individuals who identified as American Indian, multiple races or ethnicities, or other race or ethnicity.

^b^ 2-item measure of nicotine dependence, range 0-6; higher values indicate greater nicotine dependence.^24^

^c^ Includes counseling support received in person, by phone, or via web.

^d^ Assessed on a scale of 1 to 10 with higher score indicating more importance, confidence, or support.

^e^ Assessed using the 8-item Patient Health Questionnaire depression scale (range, 0-24).^27^ Higher values indicate worse symptoms.

^f^ Assessed using the 7-item Generalized Anxiety Disorder Assessment (range, 0-21). Higher values indicate more symptoms.^26^

^g^ Classified as a positive score on Alcohol Use Disorders Identification Test (3 items; range, 0-12). Positive scores were 3 or higher in women or 4 or higher in men.^25^

^h^ Smoking-related diseases are those specified in the 2014 US Surgeon General’s Report.^2^ These include neoplasms (*International Classification of Diseases, Ninth Revision* (*ICD-9*) codes 140-151, 157, 161, 162, 180, 188, 189, and 204-208), cardiovascular diseases (*ICD-9* codes: 410i414, 390-398, 415-417, 420-429, 430-438, and 440-448), respiratory diseases (*ICD-9* codes: 480-492 and 496), and perinatal conditions (*ICD-9* codes: 765, 769, and 798.0).

^i^ Circulatory includes cardiovascular, peripheral vascular, and cerebrovascular diseases.

### Use of Tobacco Cessation Treatment

At 1 and 3 months after hospital discharge, a higher proportion of TTMC participants than QL participants reported current use of any tobacco cessation treatment (1 month: 489 participants [69.3%] vs 369 participants [52.5%]; *P* < .001; 3 months: 434 participants [61.5%] vs 319 participants [45.4%]; *P* < .001), any current counseling use (1 month: 245 participants [34.7%] vs 154 participants [21.9%]; *P* < .001; 3 months: 248 participants [35.1%] vs 123 participants [17.5%]; *P* < .001), and any current pharmacotherapy (1 month: 455 participants [64.4%] vs 324 participants [46.1%]; *P* < .001; 3 months: 367 participants [52.0%] vs 264 participants [37.6%]; *P* < .001) (Table 2). At 6 months, a higher proportion of TTCM than QL participants were currently using cessation medication but not counseling support (Table 2). A respondents-only sensitivity analysis reached similar conclusions.

**Table 2.** Use of Smoking Cessation Treatments After Hospital Discharge, by Group^a^

Current use of smoking cessation treatment^b^	No. (%)		Relative risk (95% CI)	*P* value
	TTCM (n = 706)	QL (n = 703)		
**Summary measures**					
Any smoking cessation treatment^c^				
1 mo	489 (69.3)	369 (52.5)	1.32 (1.21-1.44)	<.001
3 mo	434 (61.5)	319 (45.4)	1.35 (1.23-1.50)	<.001
6 mo	318 (45.0)	287 (40.8)	1.10 (0.98-1.24)	.11
Any smoking cessation counseling^d^				
1 mo	245 (34.7)	154 (21.9)	1.58 (1.33-1.88)	<.001
3 mo	248 (35.1)	123 (17.5)	2.01 (1.66-2.43)	<.001
6 mo	124 (17.6)	125 (17.8)	0.99 (0.79-1.24)	.91
Any smoking cessation medication^e^				
1 mo	455 (64.4)	324 (46.1)	1.40 (1.27-1.54)	<.001
3 mo	367 (52.0)	264 (37.6)	1.38 (1.23-1.56)	<.001
6 mo	281 (39.8)	228 (32.4)	1.23 (1.07-1.41)	.004
**Specific treatment types**					
Any nicotine replacement product				
1 mo	445 (63.0)	300 (42.7)	1.48 (1.33-1.64)	<.001
3 mo	349 (49.4)	239 (34.0)	1.45 (1.28-1.65)	<.001
6 mo	257 (36.4)	197 (28.0)	1.30 (1.11-1.51)	<.001
Varenicline				
1 mo	19 (2.7)	24 (3.4)	0.79 (0.44-1.43)	.43
3 mo	23 (3.3)	21 (3.0)	1.09 (0.61-1.95)	.77
6 mo	14 (2.0)	25 (3.6)	0.56 (0.29-1.06)	.07
Bupropion				
1 mo	25 (3.5)	41 (5.8)	0.61 (0.37-0.99)	.04
3 mo	28 (4.0)	44 (6.3)	0.63 (0.40-1.01)	.051
6 mo	38 (5.4)	44 (6.3)	0.86 (0.56-1.31)	.48
Phone counseling from study coach				
1 mo	206 (29.2)	NA	NA	NA
3 mo	202 (28.6)	NA	NA	NA
6 mo	65 (9.2)	NA	NA	NA
Other phone counseling				
1 mo	69 (9.8)	133 (18.9)	0.52 (0.39-0.68)	<.001
3 mo	81 (11.5)	92 (13.1)	0.88 (0.66-1.16)	.36
6 mo	56 (7.9)	76 (10.8)	0.73 (0.53-1.02)	.06
In-person counseling				
1 mo	27 (3.8)	29 (4.1)	0.93 (0.55-1.55)	.77
3 mo	45 (6.4)	45 (6.4)	1.00 (0.67-1.49)	.98
6 mo	41 (5.8)	63 (9.0)	0.65 (0.44-0.95)	.02
Text message support				
1 mo	51 (7.2)	81 (11.5)	0.63 (0.45-0.88)	.006
3 mo	62 (8.8)	62 (8.8)	1.00 (0.71-1.39)	.98
6 mo	45 (6.4)	56 (8.0)	0.80 (0.55-1.17)	.25
App-based support				
1 mo	25 (3.5)	25 (3.6)	1.00 (0.58-1.72)	.99
3 mo	27 (3.8)	28 (4.0)	0.96 (0.57-1.61)	.88
6 mo	19 (2.7)	34 (4.8)	0.56 (0.32-0.97)	.03
Web-based support				
1 mo	10 (1.4)	19 (2.7)	0.52 (0.25-1.12)	.09
3 mo	13 (1.8)	26 (3.7)	0.50 (0.26-0.96)	.03
6 mo	17 (2.4)	23 (3.3)	0.74 (0.40-1.37)	.33

Abbreviations: NA, not applicable; QL, quitline electronic referral; TTCM, Transitional Tobacco Care Management.

^a^ Participants lost to follow-up or with missing data were counted as having received no treatment. A sensitivity analysis limited to responders generated the same pattern of findings.

^b^ For medication, current use was use at time of the assessment. For counseling, current use was any use since the previous assessment point.

^c^ Smoking cessation treatment included counseling or any pharmacotherapy approved by the US Food and Drug Administration.

^d^ Smoking cessation counseling could be provided by a hospital-based counselor, telephone quitline coach, or text-, app-, or web-based support. Participants could endorse more than 1 medication and counseling resource.

^e^ Smoking cessation medication included nicotine replacement products, bupropion, or varenicline.

The cessation medication most often used in both groups was NRT. Less than 10% of respondents reported using varenicline or bupropion at any follow-up (Table 2). Telephone counseling was the most common method of postdischarge behavioral support in both groups. More TTCM participants than QL participants reported having received a phone or email contact about smoking from their physician at 3 months after discharge (141 of 540 respondents [26.1%] vs 79 of 527 respondents [15.0%]; *P* < .001). Encouraging physicians to make this outreach was part of the TTCM intervention.

### Delivery of Study Interventions

The TTCM interventions were delivered to nearly all participants according to study records. One or more IVR calls were answered by 579 TTCM participants (82.4%), with a median (IQR) of 3 (1-6) of 7 scheduled IVR calls completed per person. Overall, 2335 of 4899 scheduled IVR calls (47.7%) were completed. A return counselor call was triggered at 1356 of 2335 IVR calls (58.1%), and 459 of 516 participants (88.9%) scheduled for a return call completed it. A median (IQR) of 2 (1-3) return calls were completed per participant. Participants’ self-reports of calls corroborated these records. At 1-month follow-up, 344 of 514 respondents (67.0%) rated the IVR calls as very or somewhat helpful and 237 of 268 respondents who reported having talked with a counselor (88.4%) rated counselor calls as very or somewhat helpful. At 1 month, 498 of 557 respondents (89.4%) recalled receiving NRT at discharge.

Delivery of counseling calls and NRT was lower for the QL participants. Quitlines received an EHR referral and attempted to reach 629 of 703 participants (89.5%). They reached 229 participants (32.6%) to offer services, and 169 participants (24.1%) completed at least 1 counseling call (median [IQR], 2 [1-4] calls). Participants’ self-reports of calls received corroborated quitline data. At 1-month or 3-month follow-up, 192 of 317 respondents who reported having been reached by a quitline (60.6%) rated their calls with the counselor as very or somewhat helpful. Quitlines mailed a 2- or 4-week NRT sample to 89 QL participants (12.6%). However, 300 QL participants (42.7%) reported using NRT at 1-month follow-up, indicating that quitlines were not the source of NRT for most QL participants.

### Smoking Cessation

In the longitudinal analysis including data from all 3 time points, self-reported past 7-day tobacco abstinence rates, using multiple imputation to account for missing data, were higher in the TTCM group than the QL group (RR, 1.05; 95% CI, 1.02-1.08; *P* = .001). Participants in the TTCM group reported more days of self-reported abstinence after hospital discharge than QL participants in a survival analysis (eFigure in Supplement 2).

In the cross-sectional analysis, the TTCM group had higher past 7-day tobacco abstinence rates than the QL group at 1 month (RR, 1.22; 95% CI, 1.08-1.35; *P* = .001) and 3 months (RR, 1.23; 95% CI, 1.09-1.38; *P* = .001) (Table 3). However, at 6 months, the group difference was no longer statistically significant (RR 1.13, 95% CI 0.99-1.29; *P* = .08). A sensitivity analysis classifying participants with missing data as smokers had similar findings (Table 3). Participants who reported using e-cigarettes but no other tobacco products were classified as abstinent in these analyses. Repeating the analysis counting e-cigarette–only users as smokers did not alter the findings.

**Table 3.** Tobacco Abstinence Rates After Discharge, by Group

Outcome measure	Assume missing = smoking^a^				Multiple imputation^b^			
	No. (%)		RR (95% CI)^d^	*P* value	Mean %^c^		RR (95% CI)^d^	*P* value
	TTCM (n = 706)	QL (n = 703)			TTCM (n = 706)	QL (n = 703)		
**Self-reported tobacco abstinence**								
Abstinent for the past 7 d								
e-Cigarette use allowed^e^								
1 mo	291 (41.2)	246 (35.0)	1.18 (1.03-1.33)	.02	51.5	42.3	1.22 (1.08-1.35)	.001
3 mo	276 (39.1)	218 (31.0)	1.27 (1.10-1.44)	.001	50.4	40.9	1.23 (1.09-1.38)	.001
6 mo	234 (33.1)	202 (28.7)	1.16 (0.99-1.34)	.07	43.5	38.3	1.14 (0.99-1.29)	.08
No e-cigarette use allowed^f^								
1 mo	271 (38.4)	227 (32.3)	1.19 (1.03-1.36)	.02	48.7	39.6	1.23 (1.09-1.38)	.001
3 mo	254 (36.0)	195 (27.7)	1.30 (1.12-1.50)	<.001	47.1	37.2	1.27 (1.11-1.43)	<.001
6 mo	208 (29.5)	174 (24.8)	1.19 (1.00-1.40)	.045	39.1	34.6	1.13 (0.97-1.30)	.12
Abstinent since discharge, e-cigarette use allowed^e^								
1 mo	199 (28.2)	171 (24.3)	1.16 (0.97-1.37)	.10	34.3	28.3	1.22 (1.03-1.42)	.02
3 mo	153 (21.7)	119 (16.9)	1.29 (1.04-1.58)	.02	24.9	19.2	1.30 (1.06-1.58)	.01
6 mo	120 (17.0)	91 (12.9)	1.32 (1.02-1.68)	.03	18.4	14.3	1.32 (1.02-1.67)	.03
**Biochemically confirmed tobacco abstinence^g^**								
Abstinent for the past 7 d, 6 mo	110 (15.6)	96 (13.7)	1.14 (0.88-1.46)	.31	19.9	16.9	1.18 (0.92-1.50)	.19
Abstinent since hospital discharge, 6 mo	69 (9.8)	53 (7.7)	1.27 (0.90-1.78)	.17	11.3	8.7	1.30 (0.93-1.81)	.12

Abbreviations: QL, quitline electronic referral; RR, relative risk; TTCM, Transitional Tobacco Care Management.

^a^ Participants with missing outcome data are counted as smokers in these analyses.

^b^ Imputation was conducted in 2 stages: first, impute missing survey data; second, impute missing biochemical sample results.

^c^ Self-reported abstinences were estimated from 50 multiple imputation samples, and biochemically confirmed abstinences were obtained from 929 multiple imputation samples.

^d^ Controlling for stratification factors (site, admitting diagnosis, and cigarettes per day).

^e^ Participants reporting use of e-cigarettes only but no other tobacco product are counted as abstinent for this analysis.

^f^ Requires past 7-day abstinence from all tobacco products, not only cigarettes.

^g^ Prespecified primary outcome measure: self-reported past 7-day tobacco abstinence at 6-month follow-up confirmed by saliva cotinine no more than 10 μg/L (to convert to nanomoles per liter, multiply by 5.675) or carbon monoxide no more than 9 ppm. Participants were counted as smokers in the missing counted as smoking analysis if they did not provide a biological sample or exceeded cutoffs.

**Figure 2.**
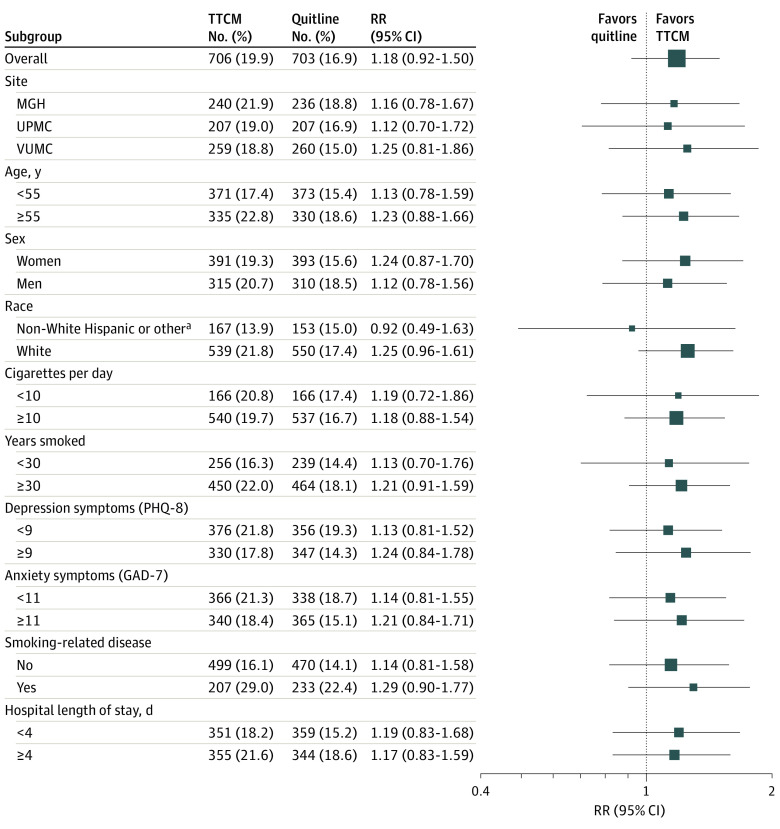
Biochemically Validated Smoking Cessation at 6 Months in Prespecified Subgroups GAD-7 indicates Generalized Anxiety Disorder Assessment (7-item version)^26^; PHQ-8, Patient Health Questionnaire (8-item version)^27^; MGH, Massachusetts General Hospital; Quitline, quitline electronic referral; TTCM, Transitional Tobacco Care Management; UPMC, University of Pittsburgh Medical Center; VUMC, Vanderbilt University Medical Center. ^a^Includes individuals who identified as Asian or other Pacific Islander, non-Hispanic Black, or other or unknown.

Among 436 participants who reported past 7-day abstinence at 6 months, 288 participants (66.1%) submitted a biological sample for confirmation, including 147 of 234 participants (63.2%) in the TTCM group and 141 of 202 participants (69.8%) in the QL group (*P* = .12). Abstinence was biochemically verified in 206 of 288 samples received (71.5%), including 110 of 147 samples (74.8%) from the TTCM group and 96 of 141 samples (68.1%) from the QL group (*P* = .20). Overall, 206 of 436 participants (47.2%) who self-reported abstinence had biochemical validation (TTCM: 110 of 234 participants [47.0%]; QL: 96 of 202 participants [47.5%]). The primary outcome measure, the proportion of participants with biochemically validated past 7-day tobacco abstinence at 6 months, did not differ significantly by group (TTCM: 19.9%; QL: 16.9%; RR, 1.18; 95% CI, 0.92-1.50; *P* = .19), although, consistent with the patterns for other outcomes, TTCM participants had a higher rate of confirmed abstinence than QL participants. Biochemically verified smoking cessation rates at 6 months did not differ significantly in any subgroup (Figure 2).

Participants in the TTCM group reported longer duration of continuous tobacco abstinence after hospital discharge than QL participants in a survival analysis (eFigure in Supplement 2). In the longitudinal analysis including data from all 3 time points, continuous tobacco abstinence rates after hospital discharge were significantly higher by self-report for the TTCM group than the QL group (RR, 1.04; 95% CI, 1.01-1.08; *P* = .01). The cross-sectional analysis showed a significant effect at 3 months (RR, 1.30; 95% CI, 1.06-1.58; *P* = .01) but not at 6 months (RR, 1.32; 95% CI, 1.02-1.67; *P* = .03) after multiple testing adjustment (Table 3). Among those reporting continuous abstinence, biochemically validated abstinence at 6 months was not significantly different between TTCM and QL participants (Table 3).

## Discussion

This large, multisite randomized clinical trial compared 2 active treatments that aimed to sustain tobacco abstinence among cigarette smokers being discharged from a hospital. The TTCM model maintained responsibility for treatment delivery within the health care system during the transition from inpatient to outpatient care, while the QL model transferred responsibility for postdischarge treatment to a community-based resource, the state quitline. Initially, the TTCM model outperformed the QL model, delivering more tobacco cessation counseling and pharmacotherapy and producing higher self-reported cessation rates during a 3-month treatment period, but the difference in abstinence rates narrowed after treatment ended and was not significantly different at 6 months. Neither biochemically validated point prevalence abstinence (the primary outcome) nor biochemically validated continuous abstinence rates differed significantly at 6 months.

We hypothesized that delivering treatment through the health care system would produce better treatment engagement and more smoking cessation than referral to a community-based quitline. Health system–based care can leverage patients’ familiarity and trust in existing caregivers and permits tobacco counselors to manage cessation medications and coordinate care with a patient’s clinical team. Previous randomized trials that compared posthospital interventions with standard care and had 6-month follow-up supported this rationale. An intervention similar to TTCM produced more cessation than did standard care in 1 trial,^10^ while referral to a quitline after hospital discharge did not increase cessation rates over standard care in 2 trials.^14,33^ In another trial, telephone counseling delivered by hospital-based research staff generated more smoking cessation than referral to a quitline.^34^

The superior treatment engagement and higher tobacco abstinence rates in the TTCM group during the 3-month treatment period may be partly attributable to a higher dose of treatment offered to TTCM participants. During the course of 3 months of treatment, TTCM offered 7 calls while QL offered 5 calls, but the large difference in counseling reported suggests that the TTCM model also offered treatment that was more accessible or acceptable to participants. Regarding NRT, TTCM provided 8 weeks in hand at discharge, while the QL offered up to 4 weeks of NRT mailed after discharge. However, study records indicate that most of the NRT use reported by QL participants did not come from the QL, suggesting that the easy access to NRT provided by the TTCM intervention encouraged more medication use.

Both TTCM and QL models assumed that the typical 3-month tobacco cessation treatment duration would suffice to sustain a quit attempt that had been prompted by hospitalization.^35^ However, abstinence rates declined more in the TTCM group than the QL group after proactive support ended at 3 months, underscoring the chronic relapsing nature of tobacco dependence. A longer treatment duration may have extended the 3-month abstinence rates in both groups. Given the efficacy of TTCM compared with QL at 3 months, extending treatment to 6 months may have sustained TTCM’s superiority over QL for long-term abstinence in this recently hospitalized population. This hypothesis warrants further study. Strengths of the study include a large, geographically diverse multisite sample, a randomized study design, study records that demonstrate fidelity of intervention delivery and corroborate participants’ self-reports, and biochemical verification of the primary smoking cessation outcome.

### Limitations

This study has some limitations. At 6 months, 26% of participants were lost to follow-up and 28% of self-reported tobacco abstinence was not biochemically verified, comparable with similar trials of smoking cessation interventions among hospitalized patients.^36^ Loss to follow-up was similar to other low-contact hospital-based trials. Rates of missing data did not differ by group, reducing concern that bias was introduced into study results. We cannot verify that a study participant was the source of remotely collected saliva and breath samples, but participants had no financial incentive to dissemble. They were reimbursed for the sample receipt, not for the test result. For co verification of self-reported nonsmoking, we used a single threshold. Carbon monoxide values were derived from either in-person monitors or smartphone-enabled remote-collection co detection devices. A small discrepancy between measurements between these devices was recently reported.^37^ Study results apply only to hospitalized smokers who plan to quit after discharge. Generalizability of QL group outcomes has the limitation that not all state quitlines offer callers unrestricted eligibility for 5 telephone counseling sessions or free NRT samples.

## Conclusions

This multisite randomized clinical trial found that delivering tobacco treatment to smokers being discharged from a hospital through the health care system or by connecting them to a community-based quitline produced no significant difference in verified tobacco abstinence rates 6 months after discharge. However, the health system–based model produced better treatment use and higher tobacco abstinence rates throughout the 3-month treatment period, but the difference waned after treatment ended. Future research should explore ways to extend postdischarge treatment to better leverage the opportunity of a hospital admission to promote smoking cessation.

## Notes

Supplement 1.
Trial Protocol and Statistical Analysis Plan

Supplement 2. **eTable.** Distribution of Missing Outcome Data by Study Group **eFigure.** Duration of Tobacco Abstinence After Hospital Discharge by Study Group

Supplement 3.
Data Sharing Statement
