# Supplementary material for: Comparative Effectiveness of Postdischarge Smoking Cessation Interventions for Hospital Patients: The Helping HAND 4 Randomized Clinical Trial
Source: JAMA Intern Med. 2022 Jun 27;182(8):814–24. doi: 10.1001/jamainternmed.2022.2300 (PMC9237801; doi:10.1001/jamainternmed.2022.2300)
Supplement: Supplement 1. — Trial Protocol and Statistical Analysis Plan [file jamainternmed-e222300-s001.pdf]

DETAILED PROTOCOL

TITLE: Comparative Effectiveness of Post-Discharge Strategies for Hospitalized Smokers

mPIs: Nancy Rigotti, MD, Hilary Tindle, MD, MPH

REGISTRATION: Clinicaltrials.gov #NCT03603496

GRANT: NIH-NHLBI #2R01-HL111821

VERSION DATE: July 30, 2020

I. BACKGROUND AND SIGNIFICANCE

Cigarette smoking is the United States' leading cause of preventable death.[1] Despite *Healthy People 2020*'s objective to reduce adult smoking prevalence to less than 12%, 15% of U.S. adults smoked in 2014.[24,25] Smoking cessation has health benefits for all smokers, even those with smoking-related diseases.[5-7]

Effective smoking cessation treatment exists but is underused. According to clinical practice guidelines from the U.S. Public Health Service (2008) and U.S. Preventive Services Task Force (2015), effective tobacco treatment consists of counseling (in-person or by phone) and pharmacotherapy with nicotine replacement (NRT), bupropion, or varenicline.[9,26] Combining counseling and medication is more effective than either alone. However, few smokers trying to quit use behavioral methods (9%) or medication (32%).[13,27] Increasing the proportion of smokers who use proven treatment when quitting would boost population quit rates.

There is a compelling rationale to intervene with hospitalized smokers. Each year, nearly 4 million smokers (8.7% of all smokers) are hospitalized. This "teachable moment" provides an opportunity for a smoker to stop tobacco use because: (1) U.S. hospitals are smoke-free, requiring smokers to temporarily abstain from tobacco; (2) Illness, especially when tobacco-related, boosts motivation to quit[14]; (3) Smokers are accessible for intervention in the hospital.

Smoking intervention started in the hospital improves tobacco abstinence, but only if contact continues after discharge. The 2012 Cochrane systematic review of 50 RCTs led by Dr. Rigotti confirmed the efficacy of hospital-initiated smoking intervention. Intervention begun in hospital increased tobacco abstinence at 6-12 month follow-up by 37% (RR 1.37, 95%CI 1.27-1.48) but <u>only</u> if intervention was sustained for >1 month after discharge.[2] Intervention was effective for smokers regardless of diagnosis. Adding NRT increased cessation rates over counseling alone (RR 1.54, 95%CI 1.34-1.79).

There is a pressing need to translate interventions proven in efficacy studies into routine hospital practice.[2,3] The main challenge is how to continue tobacco treatment after hospital discharge. Tobacco use is a chronic disease whose successful treatment requires sustained care.[15,28] Coordinating care during the transition from inpatient to outpatient care is a key component of all chronic disease management programs.[29] It is especially critical for tobacco interventions because over half of smokers resume smoking within 3 days of hospital discharge,[19,30] long before outpatient follow-up visits occur. In addition, cost and convenience are barriers to using NRT after discharge if health insurance does not cover it.[31] Our previous research identified effective strategies (rapid outreach, provision of NRT) to overcome such barriers. The proposed study refines these strategies into a more comprehensive intervention to better promote abstinence after discharge.

U.S. hospitals' interest in adopting tobacco interventions is heightened by new financial incentives for adoption. The Joint Commission, which accredits hospitals, added new tobacco measures to the National Hospital Quality Measures (NHQM) in 2012. In 2014 they were endorsed by the National Quality Forum and adopted by Medicare.[8] In 2016 Massachusetts Medicaid adopted them as required pay-for-performance measures and in 2018, psychiatric hospitals will face financial penalties for failure to implement them.[32] NHQM require hospitals to (1) document smoking status of all admitted patients, (2) offer all smokers cessation counselling and medication in the hospital, and (3) actively connect smokers to tobacco treatment (both counselling and medication) after hospital discharge.[33] Hospitals not meeting NHQM targets face financial risk (lower Medicaid/Medicare reimbursement), leading hospitals to seek efficient and effective ways to meet them. The proposed project will address hospitals' need to meet this challenge. Hospitals have additional financial incentives to offer tobacco treatment as a way to reduce patients' future health care utilization and cost if they participate in shared savings programs through accountable care organizations.[34]

An intervention's cost-effectiveness and impact on hospital readmissions are key issues for hospitals and payers. Smoking cessation is cost-saving in the long run,[35-37] but its short-term effects on health care utilization and clinical outcomes after a hospital stay are not well studied. A pre-post study in 14 Canadian hospitals found a smoking intervention to be associated with fewer readmissions by 30 days and lower

mortality by 1 year but it did not assess program costs or cost-effectiveness.[38] The proposed study will gather readmission and cost-effectiveness data to guide U.S. hospitals, health care systems, and payers.[38]

The proposed project will compare the effectiveness and cost-effectiveness of two potentially scalable, disseminable, and sustainable interventions to provide tobacco cessation treatment after hospital discharge. Each meets NHQM standards but they differ in intensity and resources required. The models are:

Personalized Tobacco Care Management (PTCM). Our prior work produced an effective and cost-effective theory-based model (*Sustained Care*) that met the new NHQMs.[39] It leveraged a novel health communication tool, computerized phone calls using interactive voice response (IVR), to reach out to smokers after discharge and link them to evidence-based treatment resources. We propose to improve the *Sustained Care* model's effectiveness by incorporating advances in health communication and health information technology and by addressing the need for health care systems to coordinate the care of chronic diseases like tobacco dependence.[27] This new model, **Personalized Tobacco Care Management (PTCM),** adds a tobacco coach to coordinate tobacco treatment, a key component of the Chronic Care Model for managing chronic diseases.[29] The coach will have access to a health communication platform that expands and builds on what we have already found effective. The new model adds text messaging capabilities to IVR and live phone calls, allowing post-discharge contacts to be tailored to smoker preferences. Additionally, being based in the health care system, the coach can leverage the electronic health record (EHR) to integrate efforts with a smoker's ongoing outpatient primary care provider (PCP) team.

Electronic referral to a quitline (eReferral) is a less intensive alternative model built for easy dissemination. It uses the EHR to link hospitalized smokers to the national network of state-based quitlines for the provision of post-discharge cessation services. Quitlines deliver smoking cessation counseling and often a sample of NRT by phone at no cost to callers.[13,14] The national network of quitlines has the potential to serve the nearly 4 million smokers hospitalized annually. Currently, to meet NHQMs, some hospitals refer smokers to a quitline via a fax referral or other method not integrated into the EHR. Recent studies found that referring hospitalized smokers to a quitline in this way did not improve quit rates over usual care, perhaps because the referral process was unreliable.[40,41] eReferral is a new technologically-advanced strategy that links hospital EHRs to quitlines in a secure, bi-directional fashion. It automates the way that hospitals refer to and get feedback from a quitline, which may improve treatment uptake by patients after discharge. Due to its low cost, eReferral is likely to be attractive to hospitals and become the future standard of care for meeting NHQMs. However, how eReferral compares to PTCM for sustaining tobacco abstinence post-discharge is unknown.

Both interventions harness technology to streamline the delivery of tobacco cessation treatment by automating intervention components and by integrating with new health information technology (i.e., certified EHRs). Yet the comparative effectiveness of eReferral and PTCM to maximize benefit for hospitalized smokers is unknown. We hypothesize that PTCM will be more effective than eReferral at (1) reaching smokers to offer post-discharge treatment, (2) engaging smokers in using post-discharge tobacco cessation treatment, and ultimately (3) producing higher rates of tobacco abstinence after hospital discharge.

Potential Impact: Strong evidence supports the efficacy of initiating tobacco cessation interventions in hospitals, but to have public health impact, this key research finding needs to be translated into routine clinical practice. The main challenge is sustaining tobacco treatment practically and economically after hospital discharge. This project compares the effectiveness and cost-effectiveness of PTCM and eReferral, two broadly disseminable models that address this challenge, satisfy NHQM standards, and thereby help translate research findings into clinical practice. A cost-effective model for hospitalized smokers could, if widely adopted, reduce population smoking rates, tobacco-related mortality and morbidity and health care costs.

PRELIMINARY WORK

Progress Report for #R01-HL11821 *(Project Period 3/7/2012-6/30/2016)*

Helping HAND 1 (HH1) RCT: An earlier grant (*#RC-1HL099668, PI: Rigotti)* supported the Helping HAND 1 (Hospital-initiated Assistance for Nicotine Dependence) RCT done at MGH (2010-2012).[70] It tested the effectiveness of the Sustained Care intervention for facilitating the delivery of post-discharge tobacco cessation treatment in 397 hospitalized smokers motivated to quit. Using IVR technology, it made 5 proactive

automated phone calls to smokers over 3 months, starting 3 days post-discharge. Smokers also received a free 30-day supply of their choice of FDA-approved cessation medication in hand at discharge (refillable twice). Each IVR call reminded smokers to stay quit and offered a call back from a hospital-based tobacco coach who provided brief counseling and medication refills. Acceptance of the IVR calls was high; a median of 4 of 5 calls was completed. Validated tobacco abstinence rates were 71% higher in the intervention group at 6 months compared to advice to use cessation medication and call a quitline (26% vs.15%, p<.01).[9] The incremental cost-per-quit was $3217.[39] This single-site study demonstrated the effectiveness of the Sustained Care intervention compared to standard care in a large hospital (Mass General Hospital, MGH).

Helping HAND 2 (HH2) RCT: The current grant *(#R01-HL11821,PI: Rigotti, site PI: Tindle*).

***Aim 1**: To test the effect of a Sustained Care intervention offered to hospitalized smokers on the use of tobacco treatment and on biochemically-validated tobacco abstinence [primary outcome measure] after hospital discharge.* HH2 aimed to enhance the scalability of the HH1 intervention by incorporating referral to a telephone quitline, an evidence-based, nationally-accessible resource offering free cessation support by telephone to U.S. smokers.[13,14] The new Sustained Care model aimed to link smokers seamlessly in one call from the IVR service to a quitline. Smokers had access to counseling and medication refills provided by a tobacco coach based at a quitline rather than at the hospital (as done in HH1). The multi-site HH2 RCT compared the new model to standard care among 1357 adult daily smokers admitted to 3 hospitals in 2 states (MGH and North Shore Medical Center in MA; University of Pittsburgh Medical Center in PA). All subjects received the same in-hospital smoking intervention. At discharge, subjects were randomized to the new Sustained Care model) or to Standard Care (passive referral to the state quitline for 3 months of counseling). Outcomes were assessed 1, 3, and 6 months post-discharge. Administrative data to assess readmission and mortality rates were collected for 12 months post-discharge. Progress Report: Recruitment was completed ahead of schedule. Outcomes were assessed in 81% (1 mo), 77% (3 mo), and 75% (6 mo) of enrollees. Sustained Care improved use of cessation treatment at 1, 3, and 6 months (p<.001). It increased self-reported quit rates at 1 month (43% vs. 32%, p<.001) and 3 months (37% vs. 30%, p=.008), but the effect waned by 6 months (31% vs. 27%, p=.09). Validated 6 month abstinence (1° outcome) did not differ by group (17% vs. 16%). In a post-hoc analysis, fewer smokers used counseling post-discharge in HH2 than in HH1. These results suggest, and participant reports corroborate, that real-time linkage from IVR calls to the quitline was cumbersome in practice.[56] These shortcomings of Sustained Care likely led to lower patient engagement in behavioral counseling in HH2 compared to HH1. The outcomes paper, in the Appendix, is in press (*Am J Prev Med, Oct 2016).* Additional manuscripts are in preparation.

***Aim 2**: To assess incremental cost-effectiveness of Sustained Care vs. Standard Care.* Progress Report: Because study arms did not differ in effectiveness, cost-effectiveness analysis could not be done.

***Exploratory aim**: To examine the interventions' effect on health and health care utilization (mortality, hospital readmissions) for 1 year after discharge.* Progress Report: We are in the process of analyzing administrative data to accomplish this aim. We will compare readmissions and mortality by study arm and by post-discharge smoking status. We will also examine the impact of cessation on quality of life measures.

Lessons learned from prior work. The Sustained Care Model has been tested in 2 RCTs that together enrolled over 1700 smokers.[39,56,70,71] Reflecting on its successes and shortcomings, we conclude that the key components of a practical, effective intervention are (1) providing a sample of cessation medication in hand at discharge to ensure immediate use, (2) repeated proactive outreach starting right after discharge and continuing for several months, and (3) easy access to a coach to provide cognitive-behavioral and motivational support and promote adherence to cessation medication. The HH2 trial suggests that achieving high rates of patient engagement in treatment during outpatient care is a key challenge, especially when accessing treatment resources such as a quitline that are outside the health care system.[56] We propose two strategies to promote engagement: (1) tailoring the frequency and format of outreach contacts to meet patients' preferences for mode of contact (phone or text), and (2) enhanced care coordination with a health system-based tobacco coach. The latter strategy expands on the role of the counselor in the HH1 trial, who engaged smokers more effectively than in the HH2 trial. Unlike the quitline-based HH2 counselor, who was clearly outside the health care system, participants viewed the HH1 counselor as a familiar contact linked to their health care and saw this as a positive attribute. Taken together, these RCTs guided the design of PTCM, an improved, innovative, and highly disseminable intervention which incorporates successful aspects of past work.

Other pilot work conducted by the research team.

Coordination of care by a tobacco coach increases use of proven quit aids. In a two-arm RCT of a proactive longitudinal care intervention for smoking among 633 low income veterans (Connect to Quit (CTQ), R01-CA141596, PI: Tindle, co-I: Rigotti), we demonstrated that a Chronic Care Model-based coordinated care intervention featuring a dedicated tobacco coordinator substantially increased the use of smoking cessation medication vs. a robust usual care condition without a dedicated coordinator (AOR 3.42, 95% CI (2.42-4.81).[72] The CTQ coordinator was based in the clinical setting, facilitated medication prescriptions via interactions with the PCP and primary care team, and documented care with notes in the EHR. This role resembles that planned for the tobacco coach in the PTCM model.

Quitline eReferral. Working with the North American Quitline Consortium (NAQC), Dr. Tindle has pioneered the development of the eReferral model[12] and demonstrated its feasibility to engage a clinical population of hospitalized smokers at all levels of readiness to quit smoking in one hospital (*Am J Prev Med* in press, see References).[11] With the standardized Health Level 7, version 3 templates[73], eReferral can be used by any EHR that is compliant with Meaningful Use Stage 2. For its interoperability and efficiency, this model has been endorsed by NAQC[12] as a potential national standard of care (*see letter of support, Bailey*). Direct methods of quitline referral such as eReferral, in which quitline staff proactively reach out to smokers, result in higher initial connection rates than less active methods.[74-79] Team members from the University of Pittsburgh and Vanderbilt University are participating in the Joint Commission Tobacco Electronic Clinical Quality Measure Task Force (Tob eCQM) to finalize and beta test strategies, including eReferral, to meet the NHQM.

II. SPECIFIC AIMS

Specific Aim: To conduct a randomized controlled comparative effectiveness trial of two strategies to promote smoking cessation in hospitalized patients: (1) Personalized Tobacco Care Management (PTCM) offering smokers 8 weeks of free nicotine replacement therapy (NRT) in hand at discharge and 7 proactive automated contacts over 3 months via interactive voice response (IVR) phone calls followed by email and/or text message and (2) eReferral, a one-time automated referral from the EHR to the state quitline at discharge. The proposed randomized controlled trial will enroll 1350 adult smokers admitted to 3 large hospital systems in 3 U.S. regions (MA, PA, TN) selected to vary in size, type, and region in order to demonstrate the intervention model's potential for dissemination. All patients will receive guideline-based tobacco cessation treatment delivered in-hospital by each site's existing Tobacco Treatment Service[23] directed by a team member (Drs. Rigotti, Tindle, Davis). At discharge, subjects will be randomly assigned to PTCM vs. eReferral and followed at 1, 3, and 6 months.

***Aim 1*: To increase tobacco abstinence among hospitalized smokers after hospital discharge.**

Hypothesis 1: PTCM, vs. eReferral, will increase the proportion of smokers with biochemically-validated 7-day point prevalence tobacco abstinence at 6 months after hospital discharge (1° outcome measure).

***Aim 2*: To increase hospitalized smokers' engagement in tobacco cessation treatment after discharge.**

Hypothesis 2: PTCM, vs. eReferral, will increase the proportion of smokers who are using smoking cessation treatment (counseling and/or medication) at 1 month and 3 months after hospital discharge.

***Aim 3*: To assess the incremental cost-per-quit, comparing PTCM and eReferral.**

Hypothesis 3: PTCM will cost more and be more effective than eReferral, but the incremental cost per quit will compare favorably to other tobacco cessation interventions (≤$5000/quit).

Exploratory Aims: Additionally, we will explore the interventions' effect on health and health care utilization (mortality and hospital readmissions, respectively) 5-years post-discharge, and assess implementation fidelity of both arms.

COVID-19 Quitline Data Specific Aims:

Aim 1: To assess the effect of the COVID-19 pandemic on current cigarette smokers' health perceptions, smoking behavior, efforts to quit smoking, and engagement in treatment resources.

Aim 2: To explore the effect of the COVID-19 pandemic on the success of cigarette smokers' attempts to quit.

Study Design: The proposed study is a 2-arm randomized controlled comparative effectiveness trial testing Personalized Tobacco Care Management (PTCM) vs. eReferral (Figure 2). 1350 smokers admitted to 3 hospitals will be randomly assigned to study arm at discharge and followed at 1, 3, and 6 months. The 1° study outcome is cotinine-validated 7-day point prevalence tobacco abstinence at 6-months (Aim 1). Analyses will also test the effect of the interventions on use of treatment (Aim 2), hospital re-admissions, and assess their cost-effectiveness (Aim 3).

Figure 2: Study Design

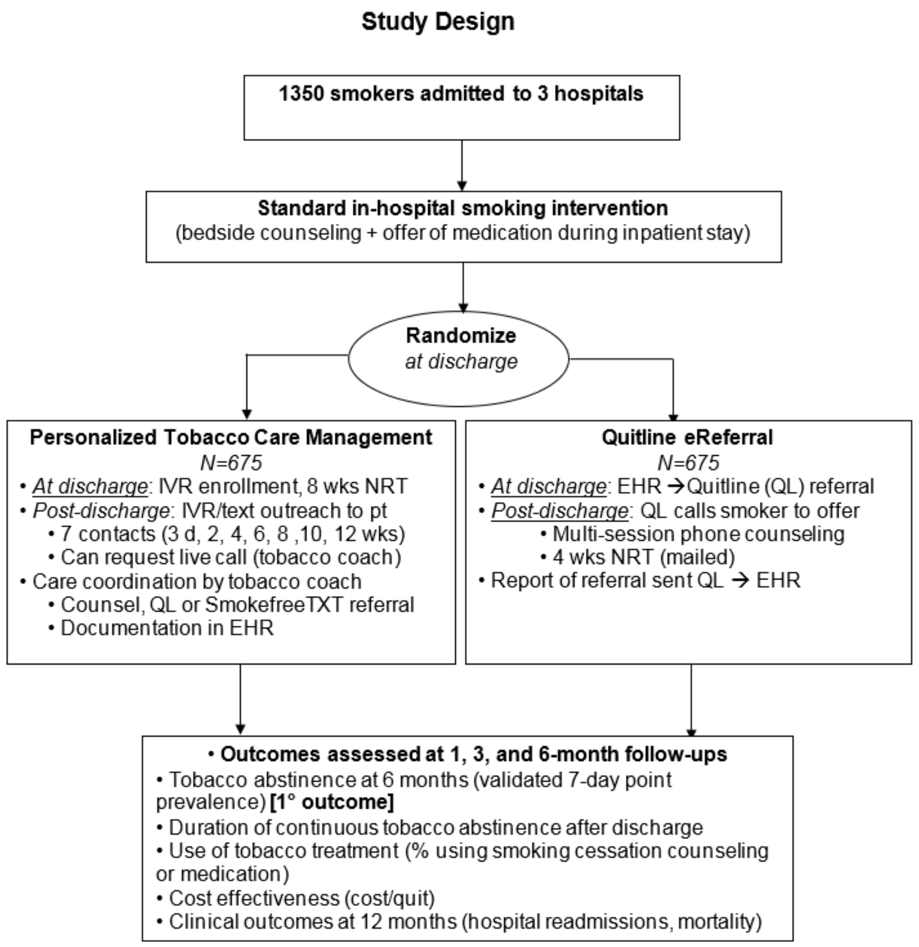

Design Rationale: We omitted a “usual care” or “no treatment” arm because the NHQM standards are likely to make hospitals reluctant to withhold tobacco treatment, and the strength of the evidence makes it ethically inappropriate to do so. Further, the ubiquity of quitlines and spread of EHRs makes it very likely that the eReferral condition will become “standard care” going forward. We decided against comparing eReferral vs. eReferral + PTCM due to concern of confusing subjects with simultaneous post-discharge outreach calls from both IVR and quitline.

Study Sites: Subjects will be recruited from 3 large private non-profit academic hospitals in 3 U.S. regions (Boston, MA, Pittsburgh, PA, Nashville, TN). **Table 1** shows the hospital characteristics. All 3 hospitals already have in place established inpatient Tobacco Treatment Services (TTS) directed by members of the study team who are their hospitals’ “tobacco champions” and who have strong support from hospital leadership (see *LOS from Drs. Slavin, Shapiro, and Mr. Edgeworth*). The in-hospital TTS care and post-discharge quitline services are uniform across sites.

Table 1: Characteristics of the 3 participating hospitals

**Hospital** *Health Care System*	**MGH** *Partners HealthCare*	**Vanderbilt** *Vanderbilt University*	**MUH / PUH** *Univ of Pittsburgh*
City/State	Boston, MA	Nashville, TN	Pittsburgh, PA
Site leader	Nancy Rigotti, MD	Hilary Tindle, MD	Esa Davis, MD
# Beds	999	1004	799
# Admissions *(2015)*	50,679	46,063	39,275
% Male	51	46	52
% White	77	71	77
Electronic health record	Epic	StarPanel*	Cerner
Quitline vendor for study	Nat Jewish Health	Nat Jewish Health	Nat Jewish Health
Smokers identified by	EHR (RN admit note)	EHR (RN admit note)	EHR (RN admit note)
Smokers identified *(2015)*	7670	6619	9309
Smokers seen *(2015)*	4471	**	4523
Smoking counselor types	Nurse, RT, HlthEd	NP, PA, RN	MSW, HlthEd
Smoking counselor FTEs	3.5	2.5	3.0
Median counseling time	9 min	**	8 min
NRT on Formulary:	Patch, gum, lozenge	Patch, gum, lozenge	Patch, gum, lozenge
Study recruitment goal	n=550	n=250	n=550

*MGH=Massachusetts General Hospital; MUH=Montefiore University Hospital; PUH=Presbyterian University Hospital; EHR=electronic health record; RT=respiratory therapist; HlthEd=health educator; *Vanderbilt switches EHR to Epic in 2018. ** Vanderbilt TTS launched in 2015. Annual # counseled and median counseling time are not known but expected to be comparable to MGH and UPMC.*

Innovation: The project's novelty is the creative way in which proven tobacco dependence treatments are adapted to a specific clinical situation (hospitalization) and packaged into two practical models for delivery by and dissemination to hospitals and health care systems. Both interventions provide proactive outreach to sustain tobacco treatment during the transition from inpatient to outpatient care and both interventions meet quality improvement milestones (NHQMs). The interventions innovatively adapt proven treatments into systems that reach smokers during the critical post discharge transition. The project leverages modern communications technology to deliver post-discharge medication and counseling, link inpatient care to community and health care-based treatment, and tailor treatment to smokers' preferences. The interventions align with the evolution of health care delivery by leveraging the EHR's capacity to deliver and track smoking cessation treatment.[43]

III. SUBJECT SELECTION

Subjects

Inclusion criteria: Adult current smokers who are admitted to a study hospital, are seen by a hospital smoking counselor, and plan to try to quit smoking after discharge. Because smokers may reduce tobacco use when ill, current smoking is defined as smoking $\geq$1 cigarette in the week before admission and $\geq$1 cigarette/day when smoking at baseline rate in the month before admission.

Exclusion criteria: Inability to give informed consent or participate in counseling due to serious cognitive or psychiatric disorder (e.g., dementia, psychosis); life expectancy <6 months; females who are pregnant or breastfeeding, medical instability precluding study participation; no reliable telephone access or inability to use telephone; non-English speaking; living in a state not covered by National Jewish Health (vendor for state quitline): Arkansas, Colorado, Idaho, Iowa, Kentucky, Massachusetts, Michigan, Nevada, New Hampshire, North Dakota, Ohio, Pennsylvania, Rhode Island, Vermont, and Wyoming (NOTES: 1) Permanent residence gleaned from site EHR; 2) Tennessee residents are included only at VUMC site.

IV. SUBJECT ENROLLMENT

Recruitment and Enrollment: Subjects will be recruited as part of MGH's, VUMC's, and MUH/PUH's standard treatment protocol for inpatients who smoke. All study sites have inpatient TTS programs that purposely resemble the evidence-based 3-step MGH program based on the successful Ottawa Model:[85] (1) Routine smoking status documentation in the EHR at admission by a nurse produces a daily list of smokers sent to the TTS. (2) A trained tobacco counselor visits these smokers to offer brief bedside counseling and medication support. (3) The counselor links the smoker to post-discharge resources by referral to the free state tobacco quitline (using older methods not integrated into the EHR, e.g., fax referral).

At the end of the hospital visit, TTS counselors will screen each smoker for study eligibility, alert eligible patients to the availability of a study offering post-discharge smoking cessation assistance, and ask about interest. The research assistant (RA) will visit a patient at the bedside to verify eligibility, explain the study in detail using a written handout that is given to the patient, and obtain verbal informed consent. Screening for eligibility will include reviewing the TTS counselor's progress note, the admission note, and contacting the patient's nurse and case manager to determine exclusion criteria. If the patient wishes to have more time to consider study participation, he or she will be given the name of the RA and study phone number. Because lengths of stay are short, it is possible that the patient will be discharged before being able to enroll in the study.

Baseline data collection will include demographic factors, admitting diagnosis, admitting service, smoking and quitting history, measures of nicotine dependence (cigarettes smoked/day in month before admission and time to first morning cigarette), intention to remain abstinent from tobacco after hospital discharge, and detailed contact information for the patient and up to 3 close contacts. Some of the data is routinely collected by the TTS counselor and entered into the TTS electronic database. It will be supplemented by additional data collected by the study research assistant at the bedside. The research assistant will use a password protected iPad, connected to the hospital's secure wireless connection, to enter baseline data directly into a RedCap project.

Assignment to Study Arm: Randomization will be stratified by (1) study site, (2) primary admitting diagnosis (cardiac vs. other), and (3) cigarettes/day (≥10, <10) to ensure that the treatment groups are balanced on these potential confounders. Prior work identified these significant independent predictors of post-discharge abstinence. After the patient enrolls, the RA will randomly assign the subject to 1 of 2 treatment conditions using the computer-generated randomization scheme created by the study statistician for the corresponding stratum. Subjects will receive a handout describing their assigned treatment condition. Intervention subjects will also receive instruction sheets on how to take nicotine patch, gum, and/or lozenge provided through the study at discharge. The PTCM and eReferral interventions begin at hospital discharge and will continue for 3 months.

Subject Payment: Subject payments are being managed by UPMC's internal/standard study subject payment system – Vincent. Vincent is the next generation of software first created in 2007 as a solution for the payment of compensation to medical study participants. Its unique features and functionality allow for the issuance of anonymous, reloadable, MasterCard-branded, instant-issue, stored value cards with multiple redemption options. Vincent allows for payments to be made in a controlled, auditable, decentralized environment with complete, centralized control and accounting to General Ledger with minimal risk and exposure to staff or clients.

At the time of enrollment, the RA will enter study subjects directly into Vincent's payment system via password protected web interface. The RA will enter the subject's name, address, and SSN (with permission). If the subject does not provide their SSN, they can still receive payment, however, their payment will be reduced by 24% for tax purposes. If the subject's card is lost or stolen a new card will be sent via USPS. The subject will be charged for the cost of the additional card by subtracting the amount ($1.25 from their payment or balance). The balance will be transferred to the new card. Subjects will be informed of these exceptions during the consent process. Once the subject is entered into Vincent payment system, they will receive a Vincent payment card, which will be loaded with payments throughout the study. All participants will receive $20 for each of the 3 follow-up telephone calls (1, 3, 6 months) that they complete, for a total compensation of $60, in addition to $20 for each of the optional qualitative phone surveys at 3- and 6-months. Participants in the PTCM condition will also receive up to 8 weeks of FDA-approved smoking cessation medication and up to 7 free smoking cessation counseling sessions at no cost. At 6 months, all participants who report that they are not smoking will be asked to provide a saliva sample as biochemical confirmation of abstinence or come in to conduct an expired CO reading (if on NRT). If the participant is unable to come in to conduct a CO, then a personal CO device (CoVita iCO Smokerlyzer) will be mailed to the participant to complete the CO reading remotely. Participants will be paid $150 when we receive the saliva sample or the expired CO reading, regardless of whether it confirms nonsmoking.

V. STUDY PROCEDURES

Prior to study enrollment, all subjects will receive standardized in-hospital smoking intervention provided at clinical services at each study site. This infrastructure ensures consistent implementation of the in-hospital treatment and provides ready access to eligible participants at all 3 sites. The PTCM and eReferral interventions begin at hospital discharge and continue for 3 months. **Table 2** summarizes how each treatment component is operationalized in each intervention.

Table 2. Operationalization of Treatment Components in the Interventions

Treatment Component	eReferral Model	PTCM Model
**Post-discharge outreach**<br>Modality<br>Frequency	Phone call<br>1 time	Automated contacts within 3 months after discharge (welcome at discharge, 3d, 2, 4, 6, 8, 10, 12wks). Contact modalities: automated IVR call, followed by automated email/text message.
**Free Counseling**<br>Services offered<br>When offered<br>Provider, location	5-call QL protocol (3 mo)<br>At initial quitline call<br>Tobacco coach (based at NJH)	Tobacco coach (based at hospital) offered at 7 automated contacts through IVR call//email/
**Free Medication**<br>Type<br>When provided	NRT x 4 wks<br>Mailed by QL after discharge	NRT x 8 wks<br>Given at discharge
**Care coordination**	Feedback reports sent QL→EHR (no dedicated coordinator)	Tobacco coach interfaces with PCP team via EHR messages; if EHR not available (outside hospital

		system), then communicate via faxed notes
**EHR integration**	Yes (link to QL)	Yes (via Coach)
**Patient choice**	n/a	IVR call followed by email/text message (patient's choice)
QL=quitline, NJH=National Jewish Health quitline, EHR = electronic health record, IVR = interactive voice response, PCP = primary care provider, SmokefreeTXT = free NCI text messaging program		

Interventions

1. Personalized Tobacco Care Management (PTCM). Adding to the components of the Sustained Care Model, PTCM will incorporate a tobacco coach based at each hospital to coordinate care with the patient and gradually transition care to the patient's outpatient primary health care team. There are three main components to the intervention:

- ***Proactive Automated Contacts (IVR Phone, Email and/or Text Message):*** PTCM aims to enhance patient engagement by adding the option of email and text messaging to the outreach done by IVR calls in HH1 and HH2 trials. An automated communication platform will generate 7 personalized, automated outbound contacts to smokers by IVR followed by their choice of email and/or text message for 3 months post-discharge (at 3 days and 2, 4, 6, 8, 10, and 12 weeks). TelASK Technologies (Ottawa, Canada), our technical partner, developed and implemented the IVR platform successfully in the HH1 and HH2 trials. Each IVR call/email/text will assess: (1) current smoking status and intention to quit, (2) current smoking cessation medication use, and (3) medication side effects. At each contact patients will have the option to request additional support from the tobacco coach; separately, some responses will trigger a call back from the coach. Criteria to trigger a live counseling call will be: (1) patient's request; (2) patients who resumed smoking after discharge but still want to quit; (3) patients who stopped using pharmacotherapy before the end of an 8-week course; (4) patients reporting medication side effects.

- ***Tobacco Coach**:* The tobacco coach is the "human face" of the intervention to the patient and performs a dual role as coordinator and counselor. In the *coordinator* role, the coach will interact with the automated technology platform that makes outbound contacts by IVR, email and/or text message, respond to patients' requests for return calls, and coordinate tobacco treatment with the patients' outpatient primary care team, using the EHR. In the *counselor* role, the coach will provide brief (10-15 min) supervised protocol-driven behavioral counseling and medication management support upon patient request. In addition, the coach will document in the EHR at study entry and intervention completion (3-months) to the patients' primary care team indicating the patient's study enrollment, interest in quitting, medication dispensed at discharge, and tailored suggestions for addressing smoking. This process ensures that every patient receives cessation support regardless of their response to IVR/email/text outreach. At any outreach contact, patients can request a call back, which will be made within 48 hours. At that call, the coach will (1) offer 10-15 minutes of protocol-driven counseling and medication support, (2) refer the patient to additional free counseling support as needed provided by local resources, phone (state quitline) or text message (enrolling the patient in the National Cancer Institute's free SmokefreeTXT program), (3) facilitate additional medication requests by documenting in the patient's encounter in the EHR and requesting additional smoking cessation medications. The tobacco coach will follow a detailed protocol to provide motivational, cognitive-behavioral smoking cessation and relapse prevention tools, tailored to the smoker's characteristics and provide medication management and adherence support, with the goal of completing a full course of cessation medication.[70] Each site's PI (a physician) or delegate will provide clinical supervision for the coach. Each coach will complete the University of MA online basic tobacco cessation training course and in-person 4-day Core Training course (or equivalent),[86] and 6 hours of observing the site's senior CTTS counseling inpatient smokers. Sites will have monthly (or as needed) case reviews to provide a forum for working through challenging clinical scenarios. Post-discharge counseling will be evaluated by the site's PI or delegate to ensure that counselors are completing relevant counseling modules, necessary database documentation, and adhering to principles of Motivational Interviewing (MI). Completion of HH4 counseling modules and HH4 database documentation will be used

to measure "what" is discussed during a counseling session. Monitoring and coding HH4 post-discharge counseling calls, using the Brief Intervention (BI) Checklist and The Motivational Interviewing Treatment Integrity (MITI) Coding Manual 4.2.1 (Moyers, et al., 2014), will be used to measure "how" post-discharge counseling is being delivered. The site's investigator or delegate will randomly select days for monitoring counseling calls, totaling 5% of their cases, and code each counseling session using the BI checklist and MITI 4. The site's investigator or delegate will also review documentation in database (DB) to ensure that the counseling module selected in the DB is consistent with the counseling content discussed during the call and that necessary database documentation is completed. Permission to audio record or listen to counseling calls will be obtained when the coach initiates the counseling call with the patient (refer to HH4 Audio Recording-Monitoring Verbal Consent). For audio recording, a small tape recorder on speakerphone or other technology, if available, will be used to tape counseling calls. No identifiers will be used, digital files will be transcribed, and tapes (or digital files) will be destroyed after the study. For real-time monitoring, the site's investigator or delegate will listen to the counseling call when it is taking place.

- ***Smoking Cessation Medication*:** At hospital discharge, smokers receive a free standard 8-week course of treatment of their choice of nonprescription NRT (patch, gum, lozenge or a combination of patch + gum or lozenge). The drug regimen can be a continuation of NRT started in the hospital or be new at discharge. Patients in the HH1 and HH2 trials had a choice of any FDA-approved cessation medication and 95% used NRT. Only NRT is used in this trial for simplicity. We include combination NRT because some but not all studies have found it to outperform single NRT. [2,87] Following a protocol and with patient input, the inpatient TTS counselor selects NRT product and dosage, which the hospital physician must approve and prescribe. NRT is delivered to the patient before discharge with written information on use and side effects. This workflow was successful in HH2. It resembles new "Meds to Beds"[88-90] programs being implemented in the study hospitals, providing an avenue for future sustainability.

2. eReferral, the second intervention, is a new technologically-advanced, automated strategy that links hospital EHRs to a quitline via a bi-directional connection[11] using Direct, a national encryption standard for securely exchanging clinical healthcare data **(Figure 3)**. Quitlines offer free cessation support (counseling and often an NRT sample) to U.S. smokers who call a toll-free number.[13,14] Proactive telephone counseling is an efficacious smoking cessation treatment method that incorporates motivational interviewing, cognitive-behavioral counseling, and relapse prevention strategies, usually in a multi-session call format.[9,91,92] For the study, the RA enrolling the patient will make the eReferral from the EHR at discharge. Quitline staff will then attempt to contact the smoker by phone within 72 hours to offer the standard services offered by the state quitline (multiple-call counseling protocol and 4 weeks NRT).[12,73] Up to 3 contact attempts are made but in practice successful connection may take several weeks. A report of the quitline referral is sent securely to the EHR where the patients' primary health care team can access it and act upon it as needed. In accordance with best practices for Federal Health IT as determined by the Office of the National Coordinator for Health Information Technology, eReferral employs standardized recommendations for message structure, content, transport, and delivery to maximize interoperability. The standardized data elements facilitate quality improvement and reporting metrics for NHQM. The quitline provider for this study, National Jewish Health (NJH), currently serves all MA and PA patients under state contract[93] and will also serve Vanderbilt study participants to ensure uniformity of eReferral across sites.

Figure 3: eReferral Process

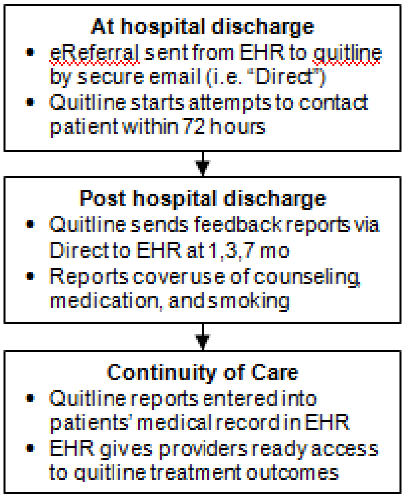

VI. BIOSTATISTICAL ANALYSIS

Data Collection

Baseline data: Each site's RA will do a baseline survey after enrollment and collect contact information for the patient and up to 3 close contacts in order to minimize loss to follow-up. To minimize burden on hospitalized subjects, some data will be obtained from hospital records and the standardized data collection

form used by smoking counselors who provide inpatient counseling at each site. Smoking counselors and RAs will be trained to ensure that they complete data forms consistently across all sites.

Blood collection for future potential genetic analyses (Vanderbilt site only): The NIH's precision medicine initiative, All of Us, aims to understand how genetics, in conjunction with a person's environment and lifestyle, can optimize prevention and treatment of illness. The GSCAN results recently published in Nature Genetics[115] identified hundreds of new genetic variants related to smoking initiation, rate, maintenance, and cessation. New information continues to become available regarding the role of genetic variation in smoking cessation and response to pharmacotherapy. The Vanderbilt University Medical Center (VUMC) site study staff will initiate a protocol to collect a blood sample on newly-enrolled and consented participants for genotyping (GWAS). The updated informed consent document thoroughly incorporates language recommended by NIH guidelines for genetic studies https://osp.od.nih.gov/wp-content/uploads/NIH_GDS_Policy.pdf. For participants who consent to provide a genetic sample, a single blood sample of approximately 15 ml (3 teaspoons) will be collected at the bedside by clinical phlebotomy/study staff.

Outcome assessment: Outcome assessments for all sites will be conducted at 1, 3, and 6 months after hospital discharge by RedCap-initiated surveys, MGH-based study staff, and mailed survey (6-month). In response to the coronavirus, a separate RedCap survey will be administered to all patients enrolled in the study who have not withdrawn or died to explore the impact of coronavirus on tobacco use behaviors and related constructs. Study participants will receive $20 uploaded to their payment card for completing this optional coronavirus survey. Study participants will select preferred method/s of contact (Participant Contact Sheet). RedCap follow-up surveys will be accessible through a password protected link sent to subjects by email and/or text message. Subjects can click on the link via computer or mobile device. Participants who do not complete the assessment by email or text, will be contacted by MGH study staff and mailed survey (6-month survey only). If participants cannot be reached, study staff will reach out to subjects' alternate contacts to find newer contact information. Subjects will receive $20 uploaded to their payment card (provided at enrollment) for each follow-up assessment completed. An additional audio-recorded qualitative survey will be offered to participants at 3- and 6-months to solicit feedback about participants' experience in participating in the study.

In addition to the HH4 Quantitative COVID Survey and HH4 Qualitative Interview (AMEs 36-38), study investigators will leverage the parent study's existing partnership with the large US state quitline operator, National Jewish Health (NJH) to address much larger population-based statewide samples. Approximately 16,000 individuals, during a one-year period, who smoke and/or vape and who are calling their state quitline in Massachusetts (MA), Pennsylvania (PA), Kentucky (KY), and Minnesota (MN) for help during the pandemic will be asked 3 questions to assess COVID-19 pandemic-related effects on their *risk perceptions*, *motivation*, and *smoking and/or vaping behavior*. These questions harmonize with the existing HH4 Quantitative COVID Survey. Survey questions will be administered by trained quitline staff as part of the standard intake process (web or phone).

Data will be maintained by NJH with permission of the Departments of Health in each state, who consider the COVID-19 related survey questions to be part of quitline care (i.e., not requiring informed consent). The HH4 team will analyze de-identified aggregated state quitline data provided by NJH. With quitline data, investigators will explore the impact of coronavirus on individuals' *risk perception, change in motivation to quit, change in tobacco use*, and *engagement in treatment resources,* as well as explore 6-month quit rates during the pandemic as compared to 1 year earlier, adjusting for any imbalance in caller characteristics.

Measures

Intervention Effectiveness: Smoking Cessation

1º Outcome measure: Verified 7-day point prevalence tobacco abstinence at 6-month follow-up

The proposed measure is self-reported abstinence from all tobacco products (including electronic cigarettes) for the past 7 days, confirmed by a saliva cotinine of $\leq$10 ng/ml or CO<9 ppm, which are consensus values.[96,97] We will obtain saliva samples by mail, an accepted method.[98] Our definition of tobacco abstinence does not allow for e-cigarette use because the FDA has deemed e-cigarettes to be tobacco products[99], but it allows for NRT use, as recommended.[96] Because of uncertainty about the risk/benefit of e-cigarettes for cessation, we will calculate a secondary tobacco abstinence measure that allows for use of e-cigarettes but no other tobacco products. (In the HH2 trial, the alternative calculation produced very small differences in abstinence rates that

did not change study results.) Because NRT use produces a false positive cotinine, the biochemical validation criterion for a patient reporting NRT use at 6 months will be an expired-air carbon monoxide measurement of ≤9 ppm, obtained at an in-person visit.[100] Subjects who self-report smoking, do not provide a saliva sample or CO measurement, whose cotinine or CO measures exceed the cut-offs, or who are lost to follow-up at 6 months will be counted as smokers.[96,97,100] Obtaining saliva samples in low-contact intervention trials, including recent studies in hospitalized smokers, is challenging.[55,101] We will offer a $150 incentive uploaded to their payment card for return of a sample, which produced a 69% return rate in HH2 (with no difference by study arm).

2º Outcome measures of tobacco use: to be calculated using standard definitions.[96]

- Self-reported 7-day and 30-day point prevalence tobacco abstinence at 1 month, 3 months, 6 months
- Sustained [repeated point prevalence] tobacco abstinence (= self-reported abstinence at 1, 3, and 6 months)
- Duration of continuous tobacco abstinence after hospital discharge (self-report)

Engagement in Tobacco Cessation Treatment

- Proportion of participants who use smoking cessation counseling or pharmacotherapy after discharge
- Duration (days) of medication use, number of counseling contacts after discharge

Definitions: Counseling can be from any evidence-based source, including clinicians, and occur in person, by phone or text message. Neither quitline registration calls nor IVR calls will be counted as counseling, but speaking to a tobacco coach at either the quitline or hospital will count. Medication includes all FDA-approved smoking cessation medications.

To succeed in promoting tobacco abstinence, post-discharge interventions must engage patients in tobacco treatment. To do so, they must reach patients after discharge, and patients must agree to use evidence-based pharmacotherapy and/or counseling. **Table 3** shows how we will measure these concepts, using a combination of patient self-reports at each assessment and records of treatment offer or delivery obtained from TelASK (IVR vendor), NJH (quitline), and SmokefreeTXT internal monitoring systems as well as from the EHR. EHR data will include (1) quitline feedback reports; (2) tobacco coach records of patient contact and coordination of care activities; (3) pharmacy records of NRT prescriptions provided by the study; (4) use of SmokefreeTXT. (Usage data from SmokefreeTXT will be visible to the tobacco coach in the EHR through the Epic-Smokefree.gov interface that is currently being programmed and will be publically available by 2017 (*personal communication with Dr. Erik Augustson, Smokefree.gov Director*). This approach utilizes the capacity of EHRs to capture data on tobacco treatment use for assessing the outcomes of interventions and illustrates how tobacco treatment interventions can be integrated into EHRs.

Table 3: Treatment engagement / use measures

**Post-discharge Interventions**	**Personalized Tobacco Care Management (PTCM)**			**eReferral to Quitline (eReferral)**		
	**Measure**	**Data sources**		**Measure**	**Data sources**	
		Surveys	Other source		Surveys	Other source
Outreach contact	% complete any IVR/Email/TXT contact<br># of IVR/ Email/TXT contacts completed	x<br>n/a	Vendor records	% reached by QL	x	QL report (EHR)
Treatment Use<br>- Medication	% given NRT at d/c<br>Duration of NRT use, other med use	x<br>x	EHR records	% given NRT at d/c<br>% sent NRT sample<br>% receive NRT	 <br>x	EHR records<br>QL report (EHR)
- Counseling	% talk to coach, # of calls completed	x<br>n/a	EHR records	% enroll in counseling	x<br>n/a	QL report (EHR)

	% referred to QL or SmokefreeTXT<br>Usage details of QL, SmokefreeTXT	x	EHR records<br>QL/TXT records	# of calls completed		

Health and health care utilization outcomes

All-cause hospitalizations, cardiac and respiratory hospitalizations, all-cause deaths over 1 year. We will compare rates of hospitalizations and mortality in the two study arms. These will be secondary, exploratory analyses. There is some evidence that hospital-initiated tobacco treatment programs can reduce readmissions by 30 days and mortality by 1 year follow-up.[38] At all follow-up surveys, we will ask subjects about subsequent hospital admissions, using standard items from the National Health Interview Survey. To corroborate patient reports, we will review administrative data from our hospitals and ask permission to review the discharge summaries from any admission to an outside hospital. Among subjects lost to follow-up, mortality will be detected via proxy contacts, by reviewing hospital records, and, if necessary, the National Death Index.

Covariates (moderators) - All done at baseline. [* = also repeated at follow-ups.]

- Sociodemographic: age, sex, education, race, ethnicity, marital status, employment, health insurance type.
- Smoking history and behavior: cigarettes/day (month before admission), intention to remain abstinent after discharge (5-point Likert scale), years smoked, past >24 hour quit attempt (ever, in past year), past use of treatment (FDA-approved meds, counseling by phone, text message, website, physician).
- Nicotine dependence: Fagerström Test for Nicotine Dependence (6-items).[102]
- Self efficacy to quit: importance, confidence in ability to quit (1 item each, 5-point Likert scale).[80,103]
- Medical history (from hospital record): tobacco-related diagnoses CHD, COPD, stroke, cancer; CVD risk factors (hypertension, diabetes, hyperlipidemia).
- Hospital course (from hospital record): primary and secondary discharge diagnoses, length of stay.
- *Depressive symptoms: 8-item Center for Epidemiologic Studies Depression Scale[104] at baseline and follow-up. Rationale: depression is common in smokers,[105] depressive symptoms predict post-discharge relapse.[106]
- *Alcohol use: AUDIT-C,[107] a 3-item survey assessing frequency, quantity, and binge drinking. Rationale: heavy alcohol use is associated with difficulty quitting.
- *Other substance use: Any past month use. Rationale: other substance use is common in smokers and may be associated with lower quit rates after treatment.

Implementation and Process Measures. Data at each site will be collected to monitor rates of study eligibility, refusal, intervention delivery, and follow-up completion, using the following RE-AIM model constructs.[108] Reach*:* % of identified smokers admitted during the study who are seen by a TTS counselor, are eligible for the study, and enroll. Effectiveness: the 1° trial outcome. Implementation: % of subjects assigned to each study arm who receive relevant components (See Table 3). These data will be obtained from TelASK, NJH, and SmokefreeTXT internal monitoring systems as well as from the EHR.

Statistical methods

Sample Size

A total sample of 1350 (675/group) will have 84% power to detect a 6.5% absolute difference in primary outcome, verified 7-day point-prevalence abstinence at 6 months, assuming rates of 16.5% (eReferral group) and 23% (PTCM group), and a 2-tailed type I error rate of .05. The rate ratio (1.39 =.23/.165) is clinically meaningful and resembles the ratio found in the Cochrane meta-analysis of smoking cessation interventions for hospitalized patients.[2] **Rationale:** *eReferral*: We conservatively estimate the abstinence rate from the HH2 trial (17% and 16%)[56]; patients in both conditions were referred to a quitline. *PTCM:* Intervention resembles that of HH1 (25% abstinence rate);[39] we conservatively estimate 23%.

VII. RISKS AND DISCOMFORTS

The potential risks to subjects include potential psychological distress from speaking with a counselor or answering questions about their smoking, and a loss of confidentiality of their healthcare data. In addition,

those patients who are randomly assigned to receive a free supply of FDA-approved smoking cessation medication may be subject to the potential side effects of using nicotine replacement therapy (NRT).

- Nicotine patch: The most common side effect is irritated skin where the patch is applied, which is commonly managed by rotating patch sites and/or applying an OTC corticosteroid cream (such as 1% hydrocortisone). In rare cases nausea, dizziness, tachycardia can occur. Nicotine patches are sold without prescription in the U.S., which is an indication of the FDA's view of their relative safety.
- Nicotine gum or lozenge: The most common side effects are sore jaw, sores in the mouth, hiccups, dyspepsia, and nausea. In rare cases nausea, dizziness, or tachycardia can occur. Many of the side effects of the gum are the result of chewing the gum improperly and can be avoided by proper technique of the gum. Nicotine gum and lozenges are sold without prescription in the U.S., which is an indication of the FDA's view of their relative safety.

Other risks:
Participants are at some risk for loss of privacy/confidentiality. If patients opt to receive phone calls on a shared phone, a family member may inquire about the nature of the phone calls. Study staff will not disclose information about participants' participation in the study to others who share a phone line with participants. They will leave the minimum amount of information necessary on participant answering machines.

There is a risk of counseling bringing up issues that are upsetting to participants. Counselors will do their best to be supportive to patients. In addition, participants will be told that they can refuse to answer any questions or terminate contact with counselors at any time.

VIII. POTENTIAL BENEFITS

Participants in this study may benefit by being connected better to effective tobacco treatment resources after hospital discharge. They will receive up to 8 weeks of FDA-approved smoking cessation medication at no cost. As a result, they may be helped to quit smoking. Generally, there is nothing better for smokers' health than stopping smoking.

IX. MONITORING AND QUALITY ASSURANCE

Data Monitoring Plan: Study staff will provide TelASK Technologies, Inc., the vendor who will conduct outreach attempts via IVR call/email/text message with patient names and relevant contact information. This information will be transferred bi-directionally through a secure FTP. During the study, TelASK will establish a secure website interface to the data management system in which study staff can view outreach and outcome results in real time.

Data quality (including visits completed during intervention window, missing data, and recruitment rates) will be monitored monthly by the database manager, Dr. Susan Regan, and systemic data problems will be reported to the PI. IVR calls, emails, and text messages provided by TelASK, Inc. will be monitored for quality assurance. In addition, the Project Manger will review 10% of the counseling progress notes for adherence to the protocol. Outcomes data will be analyzed by Dr. Yuchiao Chang, a biostatistician, using logistic regression and survival analysis. The alpha level will be set at 5%. Interim data analysis will be conducted halfway through the trial and results will be reported in the annual NIH progress report.

The principal investigator, Dr. Rigotti, and the Project manager will be responsible for monitoring the safety and effectiveness of this trial, executing the data safety and monitoring (DSM) plan, and complying with reporting requirements. Dr. Rigotti will supply a summary of the DSM report to the NIH on an annual basis as part of the progress report.

Safety Monitoring Plan: The main risks to participation in the study include the potential for psychological discomfort during counseling sessions and side effects to the FDA-approved medications. We will protect against risks of psychological discomfort by using language that is meant to offer assistance and promote health, rather than demonstrate blame or guilt. In order to protect against risk from pharmacotherapy usage, these steps will be taken:

(1) Medication is being provided only to patients in the PTCM intervention arm. These participants also receive automated telephone calls by IVR 7 times in the 3 months after hospital discharge, especially soon after discharge (at 3 days and 2, 4, 6, 8, 10, and 12 weeks). At each call, patients are specifically asked about any side effects or problems. Those who report them are advised to speak to a live tobacco counselor. Patients who request a medication refill after the initial 30 day supply are required to speak to a live counselor, who asks about medical problems before authorizing a refill of medication. Study staff will be required to report any unexpected or questionable reports of adverse effects to the study PI (Dr. Rigotti). These will be reviewed at the weekly study staff meeting. If more urgent, the PI will review immediately.

(2) The study PI (Dr. Rigotti) will review provide back up to study staff for all medical questions that arise during participant enrollment or study progress.

(3) The study PI (Dr. Rigotti) will review any serious adverse events and report them appropriately to the IRB. SAEs that represent hospital readmissions are anticipated to be frequent in this population of patients with medical illness. Each will be reviewed by the study PI to ensure that the readmission is not related to study medication.

Adverse Events Reporting: Participants who report any adverse events to the smoking counselor will be advised to discuss these side effects with their doctor. The reports of the adverse events will be summarized and reviewed by the PI on a monthly basis and reported to the IRB upon renewal of approval and to the funding agency upon continuing review. Serious adverse events will be reported immediately to the PI who will contact the participant, the participant's physician and the IRB.

X. REFERENCES

1. U.S. Department of Health and Human Services. *

The health consequences of Smoking—50 years of progress: A report of the surgeon general. .* Atlanta (GA): Centers for Disease Control and Prevention, National Center for Chronic Disease Prevention and Health Promotion (US) Office on Smoking and Health; 2014. http://www.surgeongeneral.gov/library/reports/50-years-of-progress/index.html. Accessed 11/25/15. NBK179276 [bookaccession].
2. Rigotti NA, Clair C, Munafo MR, Stead LF. Interventions for smoking cessation in hospitalised patients. *Cochrane Database Syst Rev*. 2012;5:001837.
3. Rigotti NA, Munafo MR, Stead LF. Smoking cessation interventions for hospitalized smokers: A systematic review. *Arch Intern Med*. 2008;168(18):1950-1960.
4. Centers for Disease Control and Prevention (CDC). Cigarette smoking among adults and trends in smoking cessation - united states, 2008. *MMWR Morb Mortal Wkly Rep*. 2009;58(44):1227-1232.
5. Critchley JA, Capewell S. Mortality risk reduction associated with smoking cessation in patients with coronary heart disease: A systematic review. *JAMA*. 2003;290(1):86-97. doi: 10.1001/jama.290.1.86.
6. Anthonisen NR, Skeans MA, Wise RA, et al. The effects of a smoking cessation intervention on 14.5-year mortality: A randomized clinical trial. *Ann Intern Med*. 2005;142(4):233-239.
7. van Domburg RT, op Reimer WS, Hoeks SE, Kappetein AP, Bogers AJ. Three life-years gained from smoking cessation after coronary artery bypass surgery: A 30-year follow-up study. *Am Heart J*. 2008;156(3):473-476.
8. Joint Commission. Tobacco treatment. https://www.jointcommission.org/tobacco_treatment/. Updated 2016. Accessed June 23, 2016.
9. Fiore MC, Jaen CR, Baker TB, et al. Treating tobacco use and dependence: 2008 update. clinical practice guideline. http://www.ahrq.gov/professionals/clinicians-providers/guidelines-recommendations/tobacco/clinicians/references/quickref/index.html. Updated May 2008. Accessed June 29, 2016.
10. Smith SC,Jr, Allen J, Blair SN, et al. AHA/ACC guidelines for secondary prevention for patients with coronary and other atherosclerotic vascular disease: 2006 update: Endorsed by the national heart, lung, and blood institute. *Circulation*. 2006;113(19):2363-2372. doi: 10.1161/CIRCULATIONAHA.106.174516.
11. Tindle HA, Daigh R, Reddy VK, et al. eReferral between hospitals and quitlines: An emerging tobacco control strategy . *American Journal of Preventive Medicine*. 2016;In Press.

12. North American Quitline Consortium. Guide for implementing eReferral using certified EHRs. http://c.ymcdn.com/sites/www.naquitline.org/resource/resmgr/eRef/GuideforImpeRefFeb2016.pdf. Updated 2016. Accessed June 27, 2016.
13. Shiffman S, Brockwell SE, Pillitteri JL, Gitchell JG. Use of smoking-cessation treatments in the united states. *Am J Prev Med*. 2008;34(2):102-111.
14. Miller WR, Rollnick S. *Motivational interviewing, preparing people to change addictive behavior.* New York: The Guildford Press; 1991.
15. Steinberg MB, Schmelzer AC, Richardson DL, Foulds J. The case for treating tobacco dependence as a chronic disease. *Ann Intern Med*. 2008;148(7):554-556.
16. Friedman DJ, Starfield B. Models of population health: Their value for US public health practice, policy, and research. *Am J Public Health*. 2003;93(3):366-369.
17. Evans RG, Stoddart GL. Consuming research, producing policy? *Am J Public Health*. 2003;93(3):371-379.
18. Kindig D, Stoddart G. What is population health? *Am J Public Health*. 2003;93(3):380-383.
19. Kickbusch I. The contribution of the world health organization to a new public health and health promotion. *Am J Public Health*. 2003;93(3):383-388.
20. Glouberman S, Millar J. Evolution of the determinants of health, health policy, and health information systems in canada. *Am J Public Health*. 2003;93(3):388-392.
21. Coburn D, Denny K, Mykhalovskiy E, McDonough P, Robertson A, Love R. Population health in canada: A brief critique. *Am J Public Health*. 2003;93(3):392-396.
22. McAfee T, Babb S, McNabb S, Fiore MC. Helping smokers quit--opportunities created by the affordable care act. *N Engl J Med*. 2015;372(1):5-7.
23. Regan S, Viana JC, Reyen M, Rigotti NA. Prevalence and predictors of smoking by inpatients during a hospital stay. *Arch Intern Med*. 2012;172(21):1670-1674.
24. HealthyPeiople.gov. Tobacco use objectives. https://www.healthypeople.gov/2020/topics-objectives/topic/tobacco-use/objectives. Accessed June, 23, 2016.
25. Clarke TC, Ward BW, Freeman G, Schiller JS. Early release of selected estimates based on data from the january– march 2015 national health interview survey. *National Center for Health Statistics*. 2015.
26. Siu, A for the U.S. Preventive Services Task Force. *Behavioral and pharmacotherapy interventions for tobacco smoking cessation in adults, including pregnant women: U.S. preventive services task force recommendation statement.* . *Annals of Internal Medicine*. 2015;163:622-634.
27. Centers for Disease Control and Prevention. Quitting smoking among adults- united states, 2001-2010. *MMWR*. 2011;60(44):1513-1514-1519.
28. Joseph AM, Fu SS, Lindgren B, et al. Chronic disease management for tobacco dependence. *ARCH INTERN MED*. 2011;171(21):1894-1895-1900.
29. Agency for Healthcare Research and Quality. Care coordination. http://www.ahrq.gov/professionals/prevention-chronic-care/improve/coordination/index.html. Updated 2015. Accessed June 27, 2016.
30. Rigotti NA, Arnsten JH, McKool KM, Wood-Reid KM, Pasternak RC, Singer DE. Efficacy of a smoking cessation program for hospital patients. *Arch Intern Med*. 1997;157(22):2653-2660.
31. Singleterry J, Jump Z, DiGiulio A, et al. State medicaid coverage for tobacco cessation treatments and barriers to coverage - united states, 2014-2015. *MMWR Morb Mortal Wkly Rep*. 2015;64(42):1194-1199.
32. Centers for Medicaid and Medicare Services. Hospital inpatient prospective payment system (IPPS) and long term acute care hospital (LTCH) proposed rule issues for fiscal year (FY) 2017. https://www.cms.gov/Newsroom/MediaReleaseDatabase/Fact-sheets/2016-Fact-sheets-items/2016-04-18-2.html. Updated 2016. Accessed June 27, 2016.
33. Joint Commission. **2015 flexible ORYX® performance measure reporting options**. https://www.jointcommission.org/2015_flexible_oryx_performance_measure_reporting_options/. Updated 2015. Accessed June 23, 2016.
34. Devore S, Champion RW. Driving population health through accountable care organizations. *Health Aff (Millwood)*. 2011;30(1):41-50.
35. Kahn R, Robertson RM, Smith R, Eddy D. The impact of prevention on reducing the burden of cardiovascular disease. *Circulation*. 2008;118(5):576-585.
36. Levy DE. Employer-sponsored insurance coverage of smoking cessation treatments. *Am J Manag Care*. 2006;12(9):553-562.

37. Fishman PA, Khan ZM, Thompson EE, Curry SJ. Health care costs among smokers, former smokers, and never smokers in an HMO. *Health Serv Res*. 2003;38(2):733-749.
38. Mullen KA, Manuel DG, Hawken SJ, et al. Effectiveness of a hospital-initiated smoking cessation programme: 2-year health and healthcare outcomes. *Tob Control*. 2016.
39. Rigotti NA, Regan S, Levy DE, et al. Sustained care intervention and postdischarge smoking cessation among hospitalized adults: A randomized clinical trial. *JAMA*. 2014;312(7):719-728.
40. Cummins SE, Gamst AC, Brandstein K, et al. Helping hospitalized smokers stay quit: A factorial RCT of nicotine patches and counseling. *American Journal of Preventive Medicine*. 2016(In Press).
41. Warner DO, Nolan MB, Kadimpati S, Burke MV, Hanson AC, Schroeder DR. Quitline tobacco interventions in hospitalized patients: A randomized trial. *Am J Prev Med*. 2016.
42. Henry J, Pylypchuck Y, Searcy Y., Patel V. Adoption of electronic health record systems among U.S. non-federal acute care hospitals: 2008-2015. . 2016;ONC Data Brief, no.35.
43. Boyle R., Solberg L., Fiore M. Use of electronic health records to support smoking cessation. *Cochrane Database of Systematic Reviews*. 2011;12(CD008743).
44. Tobacco control research priorities for the next decade: Working group recommendations for 2016 - 2025: Report to the NCI board of scientific advisors. . 2016.
45. Sherman SE. A framework for tobacco control: Lessons learnt from veterans health administration. *BMJ*. 2008;336(7651):1016-1019.
46. Wagner EH, Austin BT, Von Korff M. Improving outcomes in chronic illness. *Manag Care Q*. 1996;4(2):12-25.
47. Hung DY, Rundall TG, Tallia AF, Cohen DJ, Halpin HA, Crabtree BF. Rethinking prevention in primary care: Applying the chronic care model to address health risk behaviors. *Milbank Q*. 2007;85(1):69-91. doi: 10.1111/j.1468-0009.2007.00477.x.
48. Schauffler HH, McMenamin S, Olson K, Boyce-Smith G, Rideout JA, Kamil J. Variations in treatment benefits influence smoking cessation: Results of a randomised controlled trial. *Tob Control*. 2001;10(2):175-180.
49. Fiore MC, McCarthy DE, Jackson TC, et al. Integrating smoking cessation treatment into primary care: An effectiveness study. *Prev Med*. 2004;38(4):412-420. doi: 10.1016/j.ypmed.2003.11.002.
50. Smith SS, McCarthy DE, Japuntich SJ, et al. Comparative effectiveness of 5 smoking cessation pharmacotherapies in primary care clinics. *Arch Intern Med*. 2009;169(22):2148-2155. doi: 10.1001/archinternmed.2009.426.
51. Miller N, Frieden TR, Liu SY, et al. Effectiveness of a large-scale distribution programme of free nicotine patches: A prospective evaluation. *Lancet*. 2005;365(9474):1849-1854.
52. An LC, Schillo BA, Kavanaugh AM, et al. Increased reach and effectiveness of a statewide tobacco quitline after the addition of access to free nicotine replacement therapy. *Tob Control*. 2006;15(4):286-293. doi: 10.1136/tc.2005.014555.
53. Fellows JL, Bush T, McAfee T, Dickerson J. Cost effectiveness of the oregon quitline "free patch initiative". *Tob Control*. 2007;16 Suppl 1:i47-52. doi: 10.1136/tc.2007.019943.
54. Tinkelman D, Wilson SM, Willett J, Sweeney CT. Offering free NRT through a tobacco quitline: Impact on utilisation and quit rates. *Tob Control*. 2007;16 Suppl 1:i42-i46.
55. Molyneux A, Lewis S, Leivers U, et al. Clinical trial comparing nicotine replacement therapy (NRT) plus brief counselling, brief counselling alone, and minimal intervention on smoking cessation in hospital inpatients. *Thorax*. 2003;58(6):484-488.
56. Rigotti N. A., Tindle HA, Levy DE, et al. **A post-discharge smoking cessation intervention for hospital patients: Helping HAND 2 randomized clinical trial.** . *American Journal of Preventive Medicine*. 2016;In Press.
57. Curry SJ, Grothaus LC, McAfee T, Pabiniak C. Use and cost effectiveness of smoking-cessation services under four insurance plans in a health maintenance organization. *N Engl J Med*. 1998;339(10):673-679.
58. Loewenstein G, Brennan T, Volpp KG. Asymmetric paternalism to improve health behaviors. *JAMA*. 2007;298(20):2415-2417. doi: 10.1001/jama.298.20.2415.
59. National Tobacco Cessation Collaborative. Consumer demand: Design principles. *Academy fo Educational Development*.
60. Pew Research Center. **Mobile technology fact sheet**. http://www.pewinternet.org/fact-sheets/mobile-technology-fact-sheet/. Accessed June 24, 2016.

61. Lenhart A. Cell phones and american adults. http://www.pewinternet.org/2010/09/02/cell-phones-and-american-adults/;. Updated 2010. Accessed June 24, 2016.
62. Whittaker R, Borland R, Bullen C, Lin RB, McRobbie H, Rodgers A. Mobile phone-based interventions for smoking cessation. *Cochrane Database Syst Rev*. 2009;4:CD006611. doi: 10.1002/14651858.CD006611.pub2.
63. Free C, Knight R, Robertson S, et al. Smoking cessation support delivered via mobile phone text
messaging (txt2stop): A single-blind, randomised trial. *Lancet*. 2011;378:7/2/2011-49-54. www.thelancet.com.
64. Chen YF, Madan J, Welton N, et al. Effectiveness and cost-effectiveness of computer and other electronic aids for smoking cessation: A systematic review and network meta-analysis. *Health Technol Assess*. 2012;16(38):1-205.
65. Abroms LC, Boal AL, Simmens SJ, Mendel JA, Windsor RA. A randomized trial of Text2Quit: A text messaging program for smoking cessation. *Am J Prev Med*. 2014;47(3):242-250.
66. Abroms LC, Ahuja M, Kodl Y, et al. Text2Quit: Results from a pilot test of a personalized, interactive mobile health smoking cessation program. *J Health Commun*. 2012;17 Suppl 1:44-53.
67. Brendryen H, Kraft P. Happy ending: A randomized controlled trial of a digital multi-media smoking cessation intervention. *Addiction*. 2008;103(3):478-84; discussion 485-6.
68. Riley W, Obermayer J, Jean-Mary J. Internet and mobile phone text messaging intervention for college smokers. *J Am Coll Health*. 2008;57(2):245-248.
69. Smokefree.gov. About SmokefreeTXT. https://smokefree.gov/smokefreetxt-about. Accessed June 28, 2016.
70. Japuntich SJ, Regan S, Viana J, et al. Comparative effectiveness of post-discharge interventions for hospitalized smokers: Study protocol for a randomized controlled trial. *Trials*. 2012;13:124.
71. Reid ZZ, Regan S, Kelley JH, et al. Comparative effectiveness of post-discharge strategies for hospitalized smokers: Study protocol for the helping HAND 2 randomized controlled trial. *BMC Public Health*. 2015;15:1484-015-1484-0. Epub 2015 Feb 7.
72. Tindle HA, Rigotti NA, Kraemer KL, et al. Coordinated care for smoking cessation in low income veterans: The connect to quit (CTQ) trial. . *SRNT Annual Meeting Presentation*. March 3, 2016.
73. Health Level Seven International. About HL7. http://www.hl7.org/about/index.cfm?ref=footer. Accessed June 23, 2016.
74. Vidrine JI, Shete S, Cao Y, et al. Ask-advise-connect: A new approach to smoking treatment delivery in health care settings. *JAMA Intern Med*. 2013;173(6):458-464.
75. Bentz CJ, Bayley KB, Bonin KE, Fleming L, Hollis JF, McAfee T. The feasibility of connecting physician offices to a state-level tobacco quit line. *Am J Prev Med*. 2006;30(1):31-37.
76. Shelley D, Cantrell J. The effect of linking community health centers to a state-level smoker's quitline on rates of cessation assistance. *BMC Health Serv Res*. 2010;10:25-6963-10-25.
77. Bernstein SL, Jearld S, Prasad D, Bax P, Bauer U. Rapid implementation of a smokers' quitline fax referral service in an urban area. *J Health Care Poor Underserved*. 2009;20(1):55-63.
78. Sadasivam RS, Hogan TP, Volkman JE, et al. Implementing point of care "e-referrals" in 137 clinics to increase access to a quit smoking internet system: The quit-primo and national dental PBRN HI-QUIT studies. *Transl Behav Med*. 2013;3(4):370-378.
79. Adsit R, Fox BM, Tsiolis T, et al. Using the electronic healthrecord to connect primary care patients to evidence-based telephonic tobacco quitline services: A closed-loop demonstration project. *Transl Behav Med*. 2014;4(1). doi: 10.1007/s13142-014-0259-y.
80. Rigotti NA, Thorndike AN, Regan S, et al. Bupropion for smokers hospitalized with acute cardiovascular disease. *Am J Med*. 2006;119(12):1080-1087.
81. Rigotti NA, McKool KM, Shiffman S. Predictors of smoking cessation after coronary artery bypass graft surgery. results of a randomized trial with 5-year follow-up. *Ann Intern Med*. 1994;120(4):287-293.
82. Rigotti NA, Tindle HA. The fallacy of "light" cigarettes. *BMJ*. 2004;328(7440):E278-9. doi: 10.1136/bmj.328.7440.E278.
83. Tindle HA, Barbeau EM, Davis RB, et al. Guided imagery for smoking cessation in adults: A randomized pilot trial. *Complementary Health Practice Review*. 2006;11(3):165-175.
84. Tindle HA, Rigotti NA, Davis RB, Barbeau EM, Kawachi I, Shiffman S. Cessation among smokers of "light" cigarettes: Results from the 2000 national health interview survey. *Am J Public Health*. 2006;96(8):1498-1504. doi: 10.2105/AJPH.2005.072785.

85. Reid RD, Mullen KA, Slovinec D'Angelo ME, et al. Smoking cessation for hospitalized smokers: An evaluation of the "ottawa model". *Nicotine Tob Res*. 2009. doi: 10.1093/ntr/ntp165.
86. University of Massachusetts Center for Tobacco Treatment Research and Training. Training and certification. http://www.umassmed.edu/tobacco/training/. Accessed July 6, 2016.
87. Baker TB, Piper ME, Stein JH, et al. Effects of nicotine patch vs varenicline vs combination nicotine replacement therapy on smoking cessation at 26 weeks: A randomized clinical trial. *JAMA*. 2016;315(4):371-379.
88. Home infusion and pharmacy services. http://www.upmc.com/Services/home-health-care/Pages/home-pharmacy-services.aspx;. Accessed June 27, 2016.
89. McGovern P. Project brings discharge medications to patients' bedsides. http://www.upmc.com/Services/home-health-care/Pages/home-pharmacy-services.aspx;. Updated 2016. Accessed June 27, 2016.
90. A better pill. http://www.mckesson.com/blog/a-better-pill/;. Updated 2014. Accessed June 27, 2016.
91. Zhu SH, Anderson CM, Tedeschi GJ, et al. Evidence of real-world effectiveness of a telephone quitline for smokers. *N Engl J Med*. 2002;347(14):1087-1093.
92. Stead LF, Hartmann-Boyce J, Perera R, Lancaster T. Telephone counselling for smoking cessation. *Cochrane Database Syst Rev*. 2013;(8):CD002850. doi(8):CD002850.
93. North American Quitline Consortium. Quitline map. http://map.naquitline.org/. Accessed June 23, 2016.
94. Conklin CA, Robin N, Perkins KA, Salkeld RP, McClernon FJ. Proximal versus distal cues to smoke: The effects of environments on smokers' cue-reactivity. *Exp Clin Psychopharmacol*. 2008;16(3):207-214.
95. Baker TB, Japuntich SJ, Hogle JM, McCarthy DE, Curtin JJ. Pharmacologic and behavioral withdrawal from addictive drugs. *Current Directions in Psychological Science*. 2006;5(5):232-236.
96. Hughes JR, Keely JP, Niaura RS, Ossip-Klein DJ, Richmond RL, Swan GE. Measures of abstinence in clinical trials: Issues and recommendations. *Nicotine Tob Res*. 2003;5(1):13-25.
97. SRNT Subcommittee on Biochemical Verification. Biochemical verification of tobacco use and cessation. *Nicotine Tob Res*. 2002;4(2):149-159.
98. Etter JF, Neidhart E, Bertrand S, Malafosse A, Bertrand D. Collecting saliva by mail for genetic and cotinine analyses in participants recruited through the internet. *Eur J Epidemiol*. 2005;20(10):833-838. doi: 10.1007/s10654-005-2148-7.
99. Food and Drug Administration, HHS. Deeming tobacco products to be subject to the federal food, drug, and cosmetic act, as amended by the family smoking prevention and tobacco control act; restrictions on the sale and distribution of tobacco products and required warning statements for tobacco products. final rule. *Fed Regist*. 2016;81(90):28973-29106.
100. West R, Hajek P, Stead L, Stapleton J. Outcome criteria in smoking cessation trials: Proposal for a common standard. *Addiction*. 2005;100(3):299-303. doi: 10.1111/j.1360-0443.2004.00995.x.
101. Lando H, Hennrikus D, McCarty M, Vessey J. Predictors of quitting in hospitalized smokers. *Nicotine Tob Res*. 2003;5(2):215-222. doi: 10.1080/0955300031000083436.
102. Heatherton TF, Kozlowski LT, Frecker RC, Fagerstrom KO. The fagerstrom test for nicotine dependence: A revision of the fagerstrom tolerance questionnaire. *Br J Addict*. 1991;86(9):1119-1127.
103. Simon JA, Carmody TP, Hudes ES, Snyder E, Murray J. Intensive smoking cessation counseling versus minimal counseling among hospitalized smokers treated with transdermal nicotine replacement: A randomized trial. *Am J Med*. 2003;114(7):555-562.
104. Andresen EM, Malmgren JA, Carter WB, Patrick DL. Screening for depression in well older adults: Evaluation of a short form of the CES-D (center for epidemiologic studies depression scale). *Am J Prev Med*. 1994;10(2):77-84.
105. Fergusson DM, Goodwin RD, Horwood LJ. Major depression and cigarette smoking: Results of a 21-year longitudinal study. *Psychol Med*. 2003;33(8):1357-1367.
106. Thorndike AN, Regan S, McKool K, et al. Depressive symptoms and smoking cessation after hospitalization for cardiovascular disease. *Arch Intern Med*. 2008;168(2):186-191. doi: 10.1001/archinternmed.2007.60.
107. Babor TF, de la Fuente JR, Saunders J, Grant M. The alcohol use disorders identification test: Guidelines for use in primary health care. *World Health Organization*. 1992.
108. Glasgow RE, McKay HG, Piette JD, Reynolds KD. The RE-AIM framework for evaluating interventions: What can it tell us about approaches to chronic illness management? *Patient Educ Couns*. 2001;44(2):119-127.

109. An LC, Schillo BA, Kavanaugh AM, et al. Increased reach and effectiveness of a statewide tobacco quitline after the addition of access to free nicotine replacement therapy. *Tob Control*. 2006;15(4):286-293.
110. Cummings KM, Hyland A, Carlin-Menter S, Mahoney MC, Willett J, Juster HR. Costs of giving out free nicotine patches through a telephone quit line. *J Public Health Manag Pract*. 2011;17(3):E16-23.
111. Ladapo JA, Jaffer FA, Weinstein MC, Froelicher ES. Projected cost-effectiveness of smoking cessation interventions in patients hospitalized with myocardial infarction. *Arch Intern Med*. 2011;171(1):39-45.
112. Meenan RT, Stevens VJ, Hornbrook MC, et al. Cost-effectiveness of a hospital-based smoking cessation intervention. *Med Care*. 1998;36(5):670-678.
113. Hollis JF, McAfee TA, Fellows JL, Zbikowski SM, Stark M, Riedlinger K. The effectiveness and cost effectiveness of telephone counselling and the nicotine patch in a state tobacco quitline. *Tob Control*. 2007;16 Suppl 1:i53-i59.
114. Blumberg SJ, Luke JV, Ganesh N, Davern ME, Boudreaux MH, Soderberg K. Wireless substitution: State-level estimates from the national health interview survey, january 2007-june 2010. *Natl Health Stat Report*. 2011;(39)(39):1-26, 28.
115. Liu M, Jiang Y, Wedow R, et al. Association studies of up to 1.2 million individuals yield new insights into the genetic etiology of tobacco and alcohol use. Nat Genet. 2019.
