# Supplementary material for: Comparative Effectiveness of Postdischarge Smoking Cessation Interventions for Hospital Patients: The Helping HAND 4 Randomized Clinical Trial
Source: JAMA Intern Med. 2022 Jun 27;182(8):814–24. doi: 10.1001/jamainternmed.2022.2300 (PMC9237801; doi:10.1001/jamainternmed.2022.2300)
Supplement: Supplement 2. — eTable. Distribution of Missing Outcome Data by Study Group eFigure. Duration of Tobacco Abstinence After Hospital Discharge by Study Group [file jamainternmed-e222300-s002.pdf]

Supplemental Online Content

Rigotti NA, Chang Y, Davis EM, et al. Comparative effectiveness of postdischarge smoking cessation interventions for hospital patients: the Helping HAND 4 randomized clinical trial. *JAMA Intern Med*. Published online June 27, 2022. doi:10.1001/jamainternmed.2022.2300

eTable. Distribution of Missing Outcome Data by Study Group

eFigure. Duration of Tobacco Abstinence After Hospital Discharge by Study Group

This supplemental material has been provided by the authors to give readers additional information about their work.

© 2022 Rigotti NA et al. *JAMA Internal Medicine.*

eTable. Distribution of Missing Outcome Data by Study Group

**Total Sample Size**	**ALL** 1409		**TTCM** 706		**Quitline** 703	
**1-month survey**						
deceased	8	0.6%	5	0.7%	3	0.4%
survey missing	249	17.7%	131	18.6%	118	16.8%
reached	1152	81.8%	570	80.7%	582	82.8%
survey item missing	17	1.2%	7		13	
**3-month survey**						
deceased	23	1.6%	11	1.6%	12	1.7%
survey missing	310	22.0%	149	21.1%	161	22.9%
reached	1076	76.4%	546	77.3%	530	75.4%
**6-month survey**						
deceased	43	3.1%	19	2.7%	24	3.4%
survey missing	328	23.3%	167	23.7%	161	22.9%
reached	1038	73.7%	520	73.7%	518	73.7%
survey item missing	7	0.5%	3		4	
self-reported smoking	595		283		312	
**6-month bio-confirmation**						
self-reported abstinence	436	42.0%	234	45.0%	202	39.0%
relapsed	36		21		15	
sample missing	112		66		46	
sample provided	288	66.1%	147	62.8%	141	69.8%
Untestable	2		1		1	
confirmed smoking	80		36		44	
confirmed abstinence	206		110		96	
**Total missing at 6 months**	492	34.9%	256	36.3%	236	33.6%
deceased	43	3.1%	19	2.7%	24	3.4%
survey missing	328	23.3%	167	23.7%	161	22.9%
survey item missing	7	0.5%	3	0.4%	4	0.6%
sample missing	112	7.9%	66	9.3%	46	6.5%
Untestable	2	0.1%	1	0.1%	1	0.1%

© 2022 Rigotti NA et al. *JAMA Internal Medicine.*

eFigure. Duration of Tobacco Abstinence After Hospital Discharge by Study Group

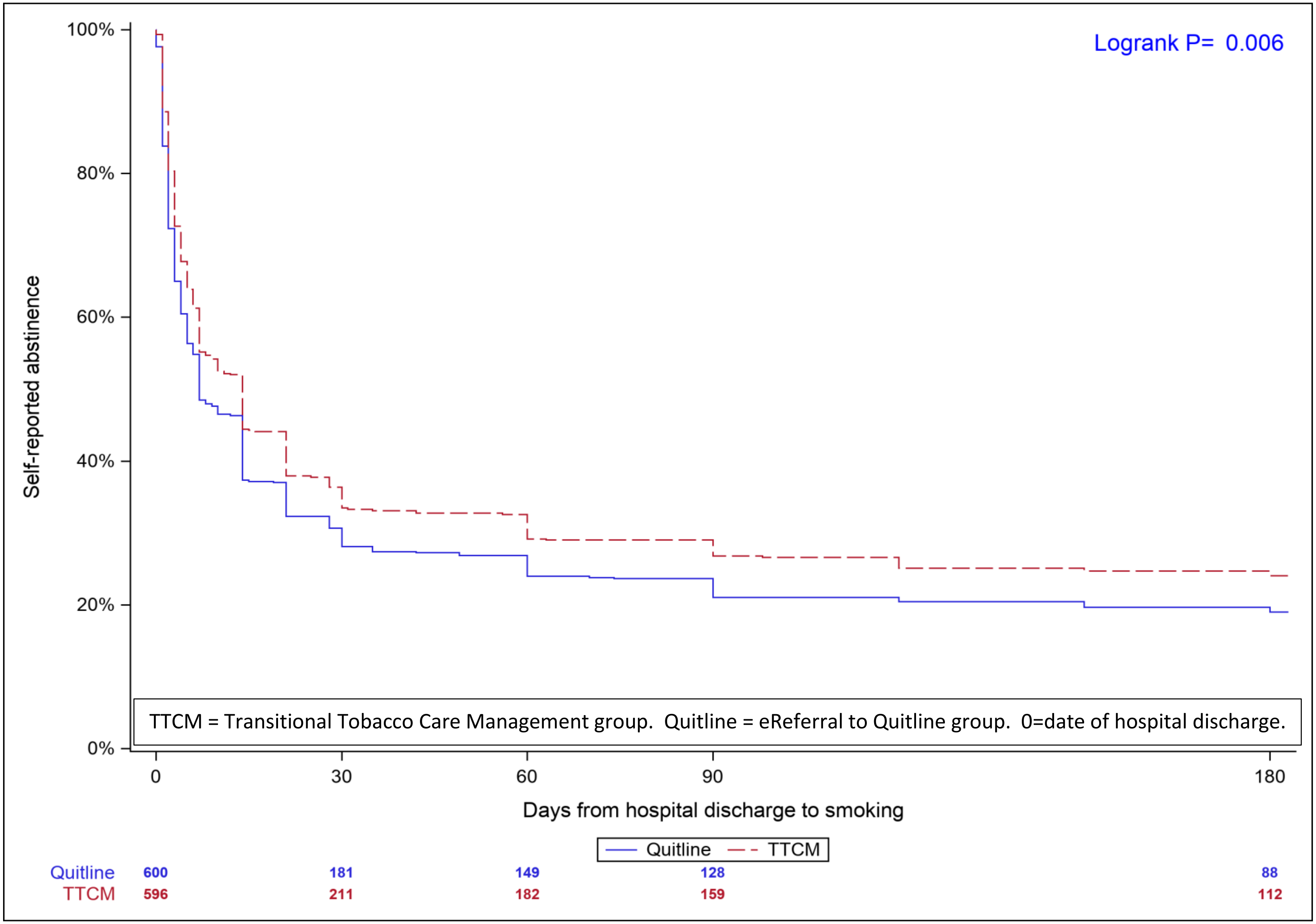

© 2022 Rigotti NA et al. *JAMA Internal Medicine.*
