# Supplementary material for: Comparative Effectiveness of Postdischarge Smoking Cessation Interventions for Hospital Patients: The Helping HAND 4 Randomized Clinical Trial
Source: JAMA Intern Med. 2022 Jun 27;182(8):814–24. doi: 10.1001/jamainternmed.2022.2300 (PMC9237801; doi:10.1001/jamainternmed.2022.2300)
Supplement: Supplement 3. — Data Sharing Statement [file jamainternmed-e222300-s00.pdf]

Data Sharing Statement

Rigotti. Comparative Effectiveness of Postdischarge Smoking Cessation Interventions for Hospital Patients. *JAMA Intern Med*. Published June 27, 2022.
doi:10.1001/jamainternmed.2022.2300

Data
Data available: Yes
Data types: Deidentified participant data
How to access data: Upon request to the lead author and after approval of analyses planned
When available: With publication

Supporting Documents
Document types: None

Additional Information
Who can access the data: Researchers whose proposed use of the data has been approved
Types of analyses: for any purpose
Mechanisms of data availability: without investigator support
Any additional restrictions: n/a
